# Action and mechanism of the colistin resistance enzyme MCR-4

**DOI:** 10.1038/s42003-018-0278-1

**Published:** 2019-01-25

**Authors:** Huimin Zhang, Mengyun Hou, Yongchang Xu, Swaminath Srinivas, Man Huang, Lizhang Liu, Youjun Feng

**Affiliations:** 1 Department of Pathogen Biology & Microbiology, Zhejiang University School of Medicine, Hangzhou, Zhejiang 310058 China; 2 Department of General Intensive Care Unit of the Second Affiliated Hospital, Zhejiang University School of Medicine, Hangzhou, Zhejiang 310058 China; 3 Carl R. Woese Institute for Genomic Biology, University of Illinois at Urbana-Champaign, Urbana, IL 61801 USA; 4 Department of Biochemistry, University of Illinois at Urbana-Champaign, Urbana, IL 61801 USA; 5 College of Animal Sciences, Zhejiang University, Hangzhou, Zhejiang 310058 China

## Abstract

Colistin is the last-resort antibiotic against lethal infections with multidrug-resistant bacterial pathogens. A rainbow coalition of mobile colistin resistance (*mcr*) genes raises global health concerns. Here, we describe the action and mechanism of colistin resistance imparted by MCR-4, a recently-identified member from the broader MCR family. We found that MCR-4 originates from the silenced variant of *Shewanella frigidimarina* via progressive evolution and forms a phylogenetically-distinct group from the well-studied MCR-1/2 family. Domain-swapping experiments further confirmed that MCR-1 and MCR-4 transmembrane and catalytic domains are not functionally-interchangeable. However, structural and functional analyses demonstrated that MCR-4 possesses a similar PE lipid substrate-recognizable cavity and exploits an almost-identical ping-pong catalysis mechanism. MCR-4 also can alleviate colistin-triggered accumulation of reactive oxygen species (ROS). Taken together, this finding constitutes a functional proof that MCR-4 proceeds in a distinct evolutionary path to fulfill a consistent molecular mechanism, resulting in phenotypic colistin resistance.

## Abstract (web summary)

Zhang, Hou, Xu, Srinivas et al. report the mechanism of colistin resistance imparted by MCR-4, which has a distinct catalytic domain but employs a similar ping-pong catalysis mechanism to that used by the well-studied MCR-1. This study provides further insights into the bacterial resistance to colistin, the last-resort antibiotic.

## Introduction

Antimicrobial resistance is a prevalent challenge to global public health. The rapid rise in severe nosocomial bacterial infections with antibiotic resistance might render only a few antibiotics like colistin^1^, despite of its nephrotoxicity and neurotoxicity^2^, ineffective in the clinical sector. This signals the importance of a global effort to understand and contain the spread of AMR^3–5^. Since colistin, a cationic cyclic-polypeptide^6^, functions as a detergent whose hydrophobic tails have the potential to insert into and disrupt the bacterial membrane bilayer, very little natural resistance has been observed historically against colistin and was limited to certain species of *Proteus*, *Neisseria*, *Serratia*, *Morganella*, and *Providencia*^7^. This has changed since the identification of a plasmid-borne mobilized colistin resistance gene (*mcr-1*) in southern China in 2015^8^. MCR-1 is a phosphoethanolamine (PEA)-lipid A transferase that adds a PEA group to the 1(4’)-phosphate of glucosamine moieties in LPS-lipid A of the bacterial outer membrane via a putative ping-pong mechanism^9–11^. This dampens the net negative charge and consequently reduces the affinity of colistin. Obviously, it is distinct from other known mechanisms for colistin resistance, like the addition of 4-amino-4-deoxy-L-arabinose to lipid A in *Salmonella enterica*^12,13^ and *Pseudomonas aeruginosa*^14^ and the glycine/diglycine modification of lipid A in *Vibrio cholerae* biotype EI Tor^15–18^. Unlike natural/intrinsic polymyxin resistance which is limited to clonal expansion, the horizontal transfer of *mcr-1*-mediated colistin resistance is rapid and can disseminate across different bacterial species. The emergence and global spread of MCR-1 is presumably attributed to the inappropriate use (over-use and/or mis-use) of polymyxin in clinical medicine^19^, livestock production^20^ and even aquaculture^21^.

In addition to the prevalent *mcr-1*, five more *mcr*-like determinants have been identified, namely *mcr-2*^22^, *mcr-3*^23–25^, *mcr-4*^26,27^, *mcr-5*^27,28^, and *icr-Mo*, an intrinsic *mcr-1*-like homolog from *Moraxella osloensis*^29^. Among them, both *mcr-1* and *mcr-3* are globally distributed^30^. In contrast, *mcr-2* and *mcr-5* are both limited to single countries, namely Belgium^31^ and Germany^28^. Similar to scenarios seen with *mcr-1*, *mcr-3*, and its variants are carried by a variety of diversified plasmids, and disseminate across multiple countries covering Asia, Europe, and North America^11^. By contrast, *mcr-4* and its variants (from *mcr-4.2* to *mcr-4.6*) are in relatively-limited similarity when compared with *mcr-1* (Supplementary Figure 1). Along with its four additional variants, *mcr-4* was only detected in *Salmonella* in Italy, 2013^26^, and in Spain and Belgium, 2015–2016^26^. In particular, a surveillance study by Wang and coauthors^27^ indicated that *mcr-4* is widespread in swine and poultry populations in China and in several cases *mcr-4* is found along with *mcr-1*, *mcr-3,* or *mcr-5*. Retrospectively, *mcr-4* was first identified on a ColE10-type plasmid that is of a broad host range and capable of replicating in a number of bacterial species. Evidently, *mcr-4* in *Salmonella* probably originates from *Shewanella frigidimarina*^26^, and often coexists with other antimicrobial determinants like *aadA*, *tetA*, and *sul*^32^, which are common in bacteria infecting aquatic animals. Given the fact that the use of colistin in aquaculture is underestimated and the number of *mcr-4*-positive cases detected is steadily rising, we hypothesize that aquatic bacteria, such as certain species of *Shewanella*, might be genetic reservoirs for *mcr-4* gene. To demonstrate this hypothesis, further experimental evidence is highly demanded in the near future.

Despite the increasing rate of identification of *mcr*-like genes, functional aspects of these additional members of the MCR family are partially understood^33^. Given the distinct phylogenetic placement of *mcr-4* to other members of this family, a complete understanding of the evolutionary origins and biochemical mechanism of MCR-4 action and its resultant colistin resistance is clearly necessary. Here, we attempt to address this gap in order to stay one step ahead of colistin resistance and to take necessary steps at a global level to contain it and to devise small molecule inhibitors to combat it.

## Results

### Molecular phylogeny of MCR-4

**Fig. 1:**
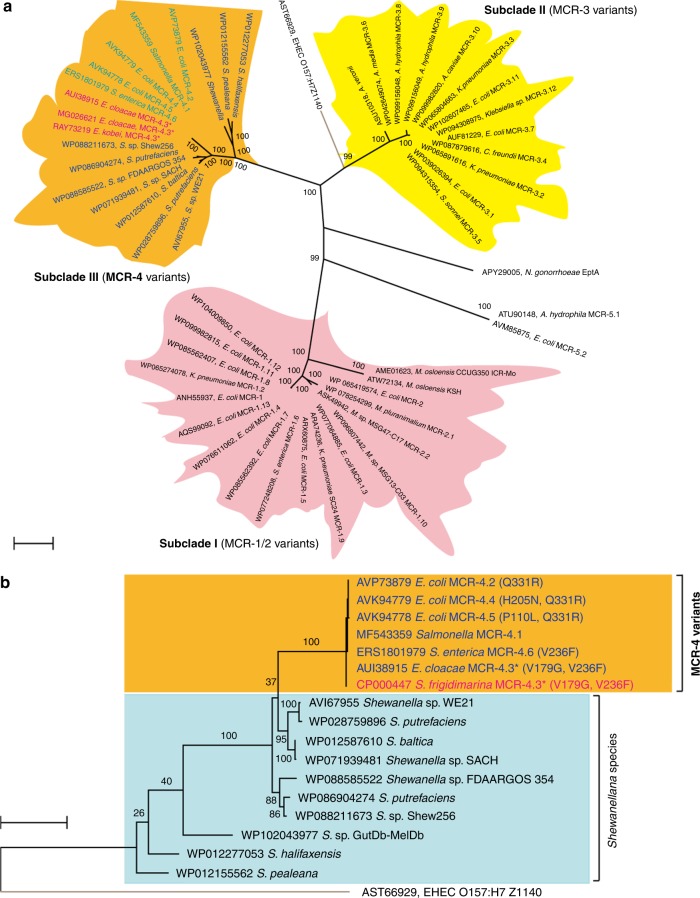
Phylogeny of MCR-4 and its variants. **a** An unrooted radial phylogram of MCR-4 and its homologs Amino acid sequences of close homologs of MCR-4, in combination with those from MCR-1/2 homologs have been included in the analysis. Three distinct phylogenetic groups refer to MCR-4 variants (in orange), MCR-3 variants (in yellow), and MCR*-*1/2 variants (in pink), respectively. In particular, a chromosomally-encoded colistin resistance determinant, *Neisseria eptA* is distinct member. Z1140 is an experimentally-verified non-functional PE transferase and acts as an internal reference in this phylogeny^9,10^. **b** Rooted phylogenetic tree of MCR-4 and its close homologs. Two distinct closely-clustered subclades have been indicated, including MCR-4 variants (highlighted in red with orange background) and MCR-4 homologs from *Shewanella* species (in a light blue background). The tree has been rooted with Z1140, an experimentally-verified non-functional PE transferase^9,10^. A protein sequence-based phylogenetic analysis of MCR-4 was constructed using the maximum likelihood method. Sequences were aligned using MUSCLE and phylogenetic trees here have been inferred using the LG model. A discrete gamma distribution was used to model evolutionary rate differences among sites with some evolutionarily invariable sites. The percentages of replicate trees in which the associated taxa are clustered in the bootstrap test (1000 replicates) is shown next to the branches. The tree is drawn to scale, with branch lengths measured in the number of substitutions per site. Protein accession numbers have been indicated in the figure. *mcr-4.3 is renamed from a duplicated *mcr-4.2*, and then found to be an inactive version^34^. The scale of bar in the phylogenetic tree is 0.20

A phylogenetic reconstruction of the MCR family generated using the maximum likelihood method depicts a broad partitioning into two groups with MCR-4 and MCR-1 occupying distinct phylogenetic positions (Fig. 1a). MCR-4 and MCR-3 (with only 34 and 45% identity to MCR-1 at protein level) are in two distinct subclades within the first group. The MCR-4 subclade is comprised of a number of its variants (MCR-4.2 to MCR-4.6), which are highly similar and have only a few substitutions (Supplementary Figure 1–2) along with MCR-4-like proteins exclusively from *Shewanella* species (Fig. 1b). MCR-3 and its variants form the second subclade within the first group. Similar to MCR-4, some of the MCR-3 genes are mobilized on plasmids and present in *E. coli* and *Klebsiella* strains while the rest are mainly intrinsic determinants of colistin resistance predominantly from *Aeromonas* species. The second broad group has three clades consisting of the MCR-2 and ICR-M families in addition to MCR-1. MCR-1 and MCR-2 share a high degree of similarity (81% identity) and are tightly clustered with each other but are a little less related to intrinsic colistin resistance enzymes from *Moraxella* (e.g., ICR-M). *Moraxella* species are broadly thought to be the current reservoir of genetic diversity for the MCR-1/2 family, with some *Moraxella* strains carrying either MCR-1 or MCR-2. Interestingly, EptA from *N. gonorrheae*, a species thought to be naturally resistant to colistin, is closer to MCR-4 than MCR-1. In fact, Z1140 which is annotated as an MCR-like enzyme, does not confer any measurable colistin resistance in the *E. coli* model^33^. Z1140 is also closer to MCR-4 than MCR-1 (Fig. 1a, b). Given that very little functional information is available for members of MCR-4 family, understanding this requires an extensive genetic and functional characterization of the members of this group.

### Gain-of-function in MCR-4 proceeds by progressive evolution

**Fig. 2:**
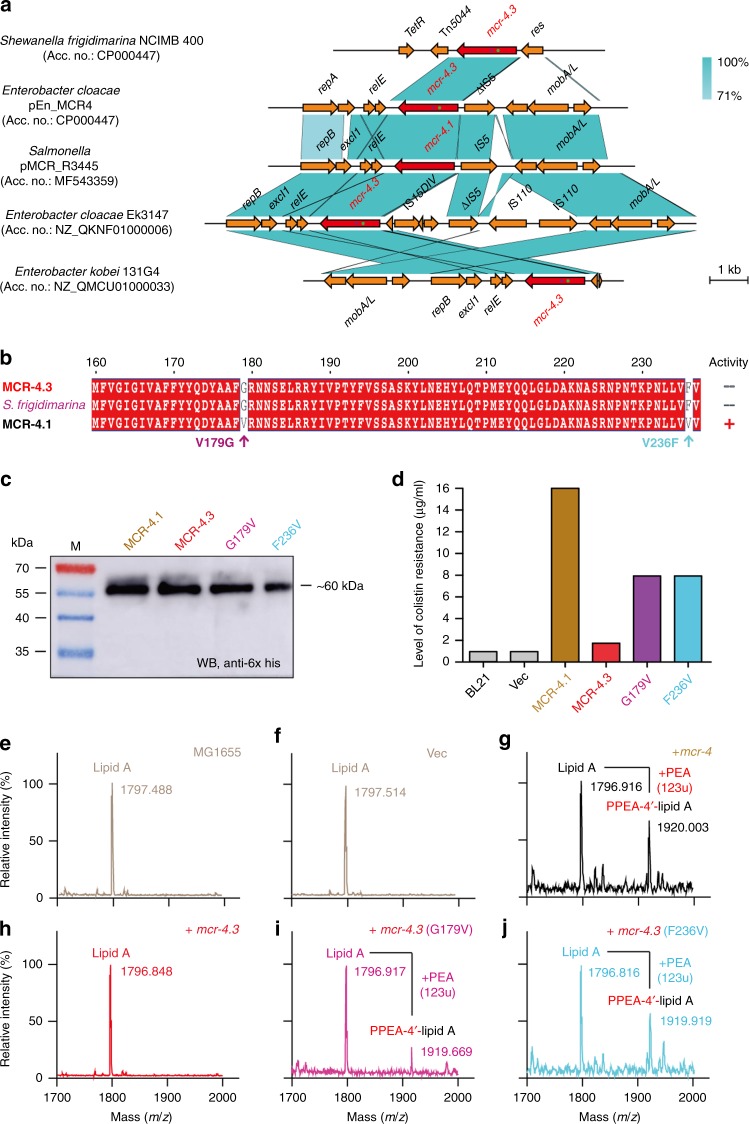
Integrative evidence that MCR-4 probably originated from the chromosomally-encoded, non-functional variant (MCR-4.3) of *Shewanella frigidimarina*. **a** Comparison of the *mcr-4.1* (*mcr-4.3*)-neighboring genetic context in the representative plasmids and/or *S. frigidimarina* chromosome Easyfig (https://omictools.com/easyfig-tool) was utilized for genomic analyses of plasmids. Colored arrows indicate ORFs and the *mcr-4* genes are highlighted in red. The shaded region depicts sequence similarity. *mcr-4.1* carried by pMCR-R3445 (Accession entry: MF543359) is the prototype for *mcr-4*, whereas *mcr-4.3* of pEn_MCR4 (Accession entry: MF061380) is determined to be an inactive variant of *mcr-4* with only two point-mutations. Of note, *mcr-4.3* was also detected on the *S. frigidimarina* chromosome. **b** Sequence alignment of a certain region in MCR-4.1 (and MCR-4.3) covering the two aforementioned point-mutations (V179G and V236F). **c** Expression analyses of *mcr-4.1*, *mcr-4.3*, and the two revertant mutants of *mcr-4.3* (G179V and F236V) in *E. coli.* The mid-log phase cultures (1 ml) were collected by spinning, dissolved in 100 μl protein loading buffer, and heated in boiling water for 20 mins. 5 μl of resulting crude extract sample was loaded into 15% SDS-PAGE for protein separation, and anti-6x His rabbit serum is the primary antibody used in Western blot. Western blot-based detection for expression of *mcr-4* variants is pre-requisite for the established relevance of bacterial viability on the condition of colistin resistance to MCR-4. M denotes pre-stained protein ladder (Thermo Scientific). The original blot is available in Supplementary Figure 15a. **d** Level of colistin resistance in *E. coli* expressing MCR-4.1, MCR-4.3, and their derivatives. No less than three independent experiments of bacterial viability were conducted with LBA plates with colistin addition. Given that bacterial growth is consistently similar, a representative result is given. MALDI-TOF mass spectrometry of lipid A species in the negative-control strains MG1655 alone (**e**) and/or with the empty vector (**f**). The addition of PEA to lipid A occurs in the strain MG1655 expressing MCR-4 (**g**), but not upon the expression of *mcr-4.3* (**h**). The two revertant mutations [G179V (**i**) and F236V (**j**)] of *mcr-4.3* partially restore its enzymatic activity in modifying the lipid A into PEA-4’-lipid A

To further address its possible origin, we conducted a BLAST search against the NCBI database using the *mcr-4* nucleotide sequence as a probe. Consequently, we noticed that (i) two *mcr-4.3*-containing contigs and/or shotgun sequences are present in *Enterobacter cloacae* complex sp. strain Ek3147 (12808 bp, Accession entry: NZ_QKNF01000006) and *Enterobacter kobei* 131G4 (7654 bp, Accession entry: NZ_QMCU01000033), respectively (Fig. 2a); and (ii) the two highly-similar ColE-type plasmids (namely pMCR_R3445 and pEn_MCR4) harbor *mcr-4.1* and/or its variant *mcr-4.3* (Fig. 2a). Obviously, the two *mcr-4.3*-harboring shotgun sequences display appreciable similarity of nucleotide sequence and genetic context to those of the two *mcr-4*-positive plasmids (Fig. 2a). Among them, pMCR_R3445 [8749 bp, Accession entry: MF543359] from *Salmonella* refers to a prototype of *mcr-4.1*-bearing plasmids, and pEn_MCR4 (8639 bp, Accession entry: MH061380) of *E. cloacae* possesses *mcr-4.3* (Fig. 2a), featuring with two point-mutations (V179G and V236F) in relative to *mcr-4.1* (Fig. 2a–b). The aforementioned two *mcr-4*-positive plasmids displayed an appreciable consistency (75% query coverage and 99% identity) in their genetic context neighboring *mcr-4.1* (and/or *mcr-4.3*). Of the 9 open reading frames (ORFs) in pEn_MCR4, a conservative operon of *relE*-*mcr-4.1* (and/or *mcr-4.3*)-IS*5* is present (Fig. 2a). More intriguingly, the plasmid-borne *mcr-4.3* was found to be a 100% match to a chromosomal DNA fragment (~1.7 kb) of *S. frigidimarina* NCIMB 400 (Accession entry: CP000447), covering a putative phosphoethanolamine transferase-encoding gene and its predicted promoter of 59 bp long (AGC TAG TAT, --10; TTA TTT, --35). This raises the possibility that *mcr-4* family originates from certain species of *Shewanella*, like *S. frigidimarina*. However, in contrast to the scenario seen with *mcr-4.1*, functional expression of *mcr-4.3* (Fig. 2c) cannot support bacterial growth of a colistin-susceptible recipient strain *E. coli* MG1655 on the condition of over 1 μg/ml colistin (Fig. 2d). Thus, we speculated that *mcr-4.1* might progressively evolve from *mcr-4.3*, an inactive form of MCR-4 predominantly existing in *S. frigidimarina*^34^. To test this hypothesis, we engineered two revertant strains of *mcr-4.3*, namely G179V and F236V (Fig. 2c, d). As predicted, the expression of both *mcr-4.3* (G179V) and *mcr-4.3* (F236V) (seen in Fig. 2c) allowed the colistin-susceptible strain *E. coli* MG1655 to grow on LBA plates with up to 8 μg/ml of colistin (Fig. 2d). MALDI-TOF mass spectrometry of lipid A pools isolated from *E*. *coli* MG1655 with/without *mcr-4* variants further validated that no detectable modification of lipid A, PEA-4’-lipid A, is seen in the *mcr-4.3*-expressing *E. coli* (Fig. 2h). This is almost identical to those of the negative-control strains (Fig. 2e, f). Also, it constitutes metabolic evidence that the enzymatic activities of MCR-4.1 (Fig. 2g) and its two revertants of MCR-4.3 [G179V (Fig. 2i) and F236V (Fig. 2j)] appear in the transfer of PEA to the suggestive 4’-phosphate position of lipid A moieties. Given the integrated evidence presented above, we believe that functional gain of MCR-4 is proceeds via a progressive evolution from its silenced version, MCR-4.3, exclusively present in *S. frigidimarina* NCIMB 400.

### Action of MCR-4 catalysis

**Fig. 3:**
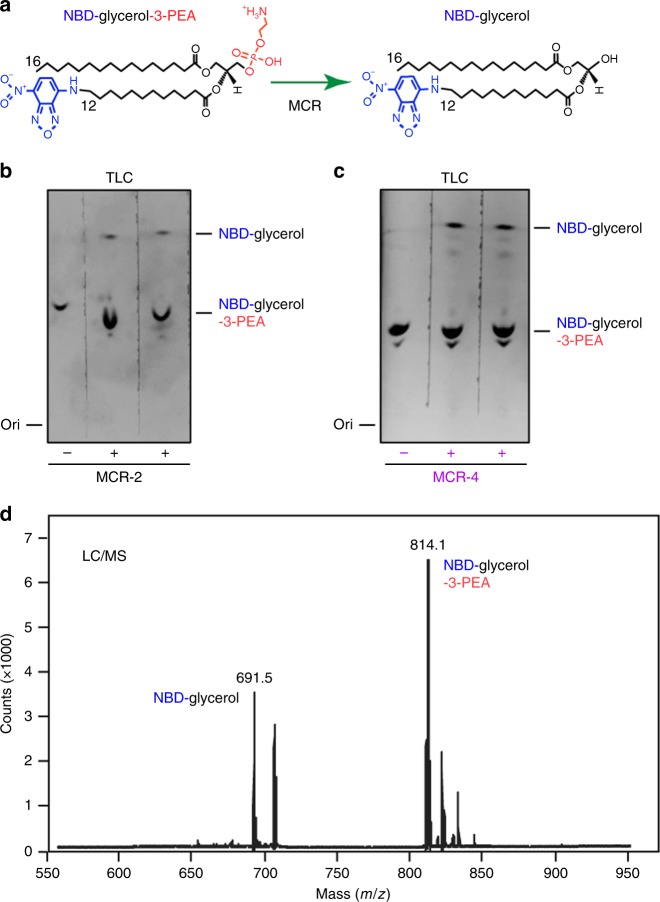
Scheme for enzymatic action of MCR-4. **a** A working model for the chemical reaction catalyzed by MCR family enzymes. This was adapted from our recent proposal^9,10^ with minor changes. In this model, MCR enzymes including MCR-4 removes the phosphoethanolamine (PEA) moiety from its alternative substrate NBD-glycerol-3-PEA, giving a putative intermediate of MCR-4-bound PEA and a final product of NBD-glycerol. NBD is indicated in blue, whereas PEA is highlighted in red. Thin layer chromatography (TLC)-based detection of enzymatic activities of MCR-2 (**b**) and MCR-4 (**c**) protein in the conversion of NBD-glycerol-PEA lipid substrate into NBD-glycerol For detection via TLC, the fluorescence signal of NBD-glycerol-3-PER (and/or NBD-glycerol) was captured under Epi blue light (455–485 nm) and a corresponding filter by the ChemiDoc MP imaging system (BioRad, CA, USA)^60^. **d** LC/MS analyses of MCR-4-mediated reaction products of NBD-glycerol-3-PEA. The spectrum of the substrate for MCR-4, NBD-glycerol-3-PEA appears at m/z of 814.1, whereas NBD-glycerol, the product of NBD-glycerol-3-PEA removing PEA (m/z, 123), is present at m/z 691.5

MCR-4 is predicted to contain two distinct domains connected by a flexible linker (Supplementary Figures 1a, b). At the N-terminus, an integral five α-helix transmembrane domain (Supplementary Figure 1a) anchors this MCR-4 protein to the inner membrane. It is connected to a periplasmic catalytic domain (Supplementary Figures 1 and 3) which is responsible for modifying Lipid A groups through the addition of a PE group in a two-step reaction. This is consistent with observations made in other MCR enzymes. First, an N-terminally hexa-histidine tagged MCR-4 was purified to homogeneity as a 66.27 kDa protein and verified by peptide mass fingerprinting with 70.31% sequence coverage (Supplementary Figure 3). Then, to visualize the reaction mechanism in vitro (Fig. 3), the purified enzyme was incubated with a fluorescent substrate NBD-Glycerol-3-PEA (Fig. 3a–d and Supplementary Figures 3-6). The reaction mixture was separated by thin layer chromatography and visualized under blue light (Fig. 3c). MCR-4 is observed to cleave the PEA group from NBD-Glycerol-3-PEA releasing NBD-Glycerol. The loss of the PE group allows NBD-glycerol to migrate faster on a TLC (Fig. 3 and Supplementary Figure 6). This production of NBD-glycerol was also verified by mass spectrometry (Fig. 3d). A similar, albeit weaker, result is observed with MCR-2 (Fig. 3b) clearly demonstrating that both enzymes can catalyze the removal of the PE group from a lipid substrate in a similar manner. This is also identical to previous observations with MCR-1 and EptA. The PE group released is hypothesized to be covalently attached to the enzyme as a first step of a ‘ping-pong’ reaction mechanism, where the PE group is then transferred to lipid A in a second step, generating PPEA-4’-lipid A (Supplementary Figure 3c). This mechanism seems to be shared across the MCR family regardless of their evolutionary placement.

### Functional definition of MCR-4 colistin resistance

**Fig. 4:**
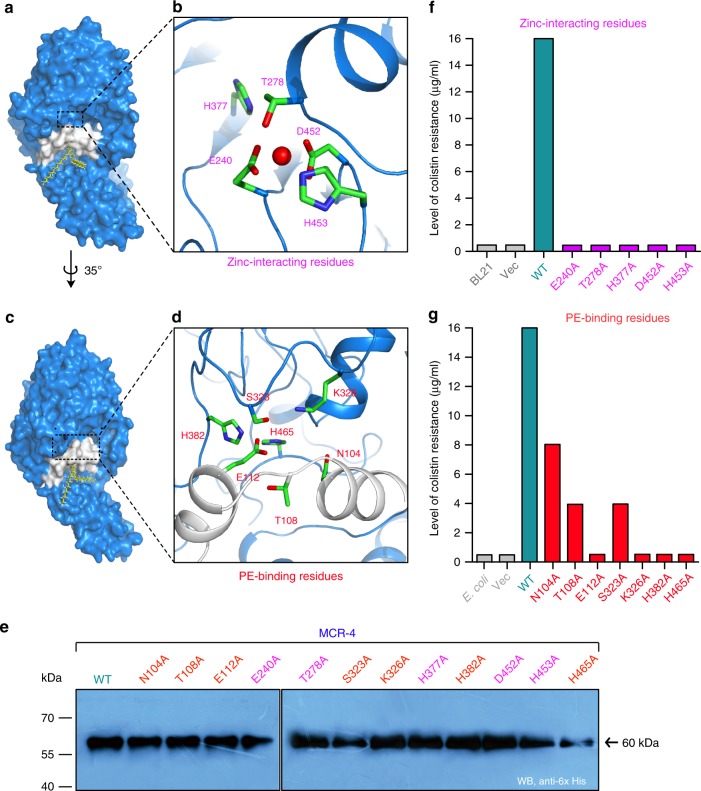
Structure and function studies of MCR-4 colistin resistance. **a** Surface structure of MCR-4 with the PE lipid substrate-interactive cavity. **b** An enlarged illustration for a five residue-containing, Zn^2+^-binding motif. The five residues in Zn^2+^-binding motif of MCR-4 refer to E240, T278, H377, D452, and H453, respectively. **c** Surface illustration of MCR-4 rotated counter-clockwise (35°). **d** An enlarged view of the seven residue-containing domain involved in binding of MCR-4 the PE lipid substrate. The seven residues denote N104, T108, E112, S323, K326, H382, and H465, respectively. **e** Use of western blotting to assay the expression of MCR-4 and its 12 point-mutants in *E. coli*. The original blot is seen in Supplementary Figure 15b. **f** Site-directed mutagenesis analyses for the Zn^2+^-binding motif of MCR-4 in the context of colistin resistance using the colistin susceptibility tests. **g** Colistin susceptibility-based dissection of the PE-interactive residues of MCR-4 It is a representative result from three independent experiments

Given the evolutionary divergence of MCR-4 from the MCR-1/2 family, it is important to evaluate the structural and functional mechanism of the MCR-like enzymes (Supplementary Figure 5). A model of MCR-4 was obtained using the X-ray crystal structure of Neisseria EptA complexed with the detergent DDM (Supplementary Figures 5b–c). Since DDM is not the PE physiological substrate of PEA transferases, we utilized molecular docking to analyze the interaction between the modeled MCR-4 structure and the physiological substrate PE (Supplementary Figure 5a–c). The head group of the PE substrate docked well into MCR-4 (Supplementary Figure 5d), and four putative residues (namely N104, T278, H382, and H465, in Supplementary Figure 5e) were clearly visualized in the ligplot+ diagram of docked PE head group into MCR-4. This was consistent with a full glimpse of the PE cavity illustrated by structural superposition of MCR-4 (Supplementary Figure 5c) with EptA (Supplementary Figure 5b). Indeed, the PE head group (Supplementary Figures 5a, d and e) is seen to be perfectly accommodated within a recently-defined substrate binding cavity seen in MCR-1/2/3 and ICR-Mo that forms part of the active site pocket (Fig. 4a–d and Supplementary Figures 5b-c)^9,10,29,33^. This includes residues N104, T108, E112, S323, K326, H382, and H465 (Fig. 4c-d). A single zinc atom is also found above this pocket with five metal-interacting residues being predicted (Fig. 4a) including E240, T278, H377, D452, and H453. The presence of zinc was confirmed via inductively coupled plasma mass spectrometry in purified MCR-4 protein preparations (Supplementary Figures 3–5). A similar substrate- and the zinc-binding site has been observed with MCR-1/2^9,10^, ICR-Mo^29^, and MCR-3^33^, suggesting a strong evolutionary conservation of the active site cavity between diverse members encoding colistin resistance.

To test the necessity of individual residues, site-directed alanine mutants of MCR-4 were constructed and their ability to confer resistance to colistin was tested in a colistin-sensitive *E. coli* background (susceptibility of <0.5 μg/ml of colistin). Wild-type MCR-4 expressed from a plasmid conferred resistance to >16 μg/ml of colistin while mutations in any of the five zinc binding residues completely rendered MCR-4 non-functional (Fig. 4f). Similarly, mutants E112A, K326A, H382A, and H465A, implicated in substrate interaction, were unable to confer any appreciable colistin resistance (Fig. 4g) indicating the critical nature of these interactions for substrate binding. However, three of the alanine substitution mutants of MCR-4 retained partial activity, namely N104A [8 μg/ml], T108A [4 μg/ml] and S323A [4 μg/ml] (Fig. 4g). The expression level of all 12 mutants was comparable to that of the wild-type in *E. coli* (Fig. 4e).

The functionality of the mutants was further evaluated in vivo by examining their ability to modify the Lipid A species of a susceptible *E. coli* MG1655 host. Lipid A*-*LPS was purified from strains expressing these mutants (Supplementary Figure 7) and subjected to MALDI-TOF MS. Wildtype cells showed a predominant peak at m/z 1796.85 corresponding to hexa-acylated and doubly-phosphorylated lipid A (Supplementary Figures 8a–b). When *mcr-4* is expressed, a new peak at m/z 1920.14 is observed (Supplementary Figure 8c) that corresponds to a PPEA modification (123 Da) of lipid A. This is also observed for the three mutants (N104A, T108A, and S323A) that retain partial activity (Supplementary Figures 8d, 8e, and 8i). All other mutants retain wildtype lipid A species (Supplementary Figures 8f–h and 8j-o) indicating their inability to modify the lipid A on LPS and their relative importance of these residues in substrate binding. These in vivo data together define a functional active site cavity that accommodates both the substrate and metal atom required for maintaining effective catalytic activity.

### Inter-domain relationships amongst MCR-4 and MCR-1/3

**Fig. 5:**
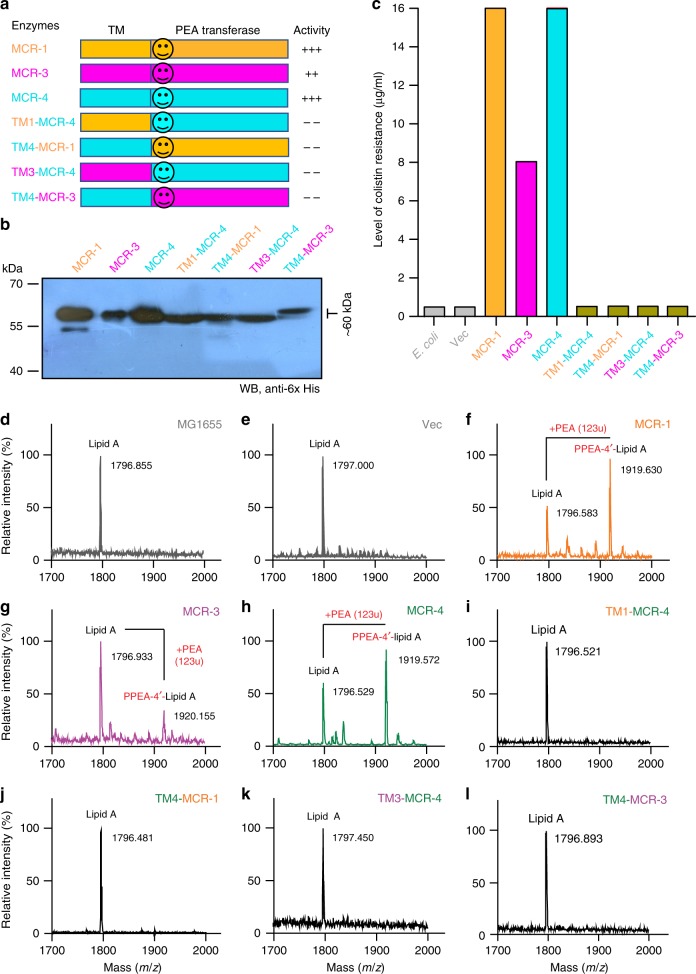
Domain-swapping analyses of three MCR-like proteins (MCR-1, MCR-3 and MCR-4). **a** Scheme for domain-swapped constructs between MCR-4 and MCR-1/3 The smiley-face refers to a linker between the transmembrane region and the catalytic domain of PEA transferase. **b** Western blot assays for the expression of *mcr-4* and its mosaic versions in *E. coli* The original blot is provided in Supplementary Figure 15c. **c** Comparison of bacterial viability of *E. coli* expressing *mcr-4* and its hybrid derivatives on the LBA plates with varied level of colistin It is a representative result from three individual assays of colistin resistance. **d**, **e** MALDI-TOF MS spectrum of the LPS-lipid A species isolated from the colistin-susceptible strain *E. coli* MG1655 with or without the empty vector pBAD24 MALDI-TOF MS profile of the LPS-lipid A species from the two positive control strains, *E. coli* MG1655 with MCR-1 (**f**) and/or MCR-3 (**g**). **h** MS evidence that MCR-4 transfer PEA to lipid A (m/z, 1796.583), producing the PPEA-4’-lipid A (m/z, 1919.630) The four domain-swapped versions of MCR-4 and MCR-1/3 that fail to render the recipient *E. coli* strain MG1655 resistant to colistin included TM1-MCR-4 (**i**), TM4-MCR-1 (**j**), TM3-MCR-4 (**k**), TM4-MCR-3 (**l**), respectively

The transmembrane and catalytic domains are observed to be interchangeable between closely-related MCR proteins like MCR-1 and MCR-2^9,10,35^. Given the recent identification of MCR-4 and its distinct phylogenetic placement, the importance and inter-compatibility of its domains with other members of the MCR family was investigated. Four chimeric proteins were engineered by swapping domains between MCR-1, MCR-3, and MCR-4 (Fig. 5a). Exchanging the TM domain of MCR-4 with that of either MCR-1 or MCR-3 renders the enzyme inactive (Fig. 5c) and unable to confer colistin resistance to sensitive *E. coli*. A similar result is observed when the TM domain from MCR-4 is fused with the catalytic domains of either MCR-1 or MCR-3 (Fig. 5c). Though the incompatibility between MCR-4 and MCR-1 is expected, it is observed that the domains of MCR-3 and MCR-4 are incompatible as well. This indicates that despite being equally distant from MCR-1 from a phylogenetic standpoint, MCR-3 and MCR-4 are fundamentally different from each other as well. All expression of these proteins was confirmed via western blotting (Fig. 5b). When tested for their ability to function in vivo, these results are mirrored (Figs 5d–i). This was evaluated by purifying lipid A-LPS from *E. coli* MG1655 strains (Supplementary Figure 9) and subjecting them to MALDI-TOF MS. While wild-type MCR-1, MCR-3, and MCR-4 can effectively add a PE group to lipid A species (m/z, 1919.3; in Fig. 5f–h), the hybrid constructs show the same lipid A species as the wildtype and vector control (m/z, 1796.855; in Figs 5d, e and 5i–l). This suggests that the inter-domain relationships are important and distinct between these different MCR proteins and crucial in maintaining their catalytic activity.

### Physiological roles of MCR-4

**Fig. 6:**
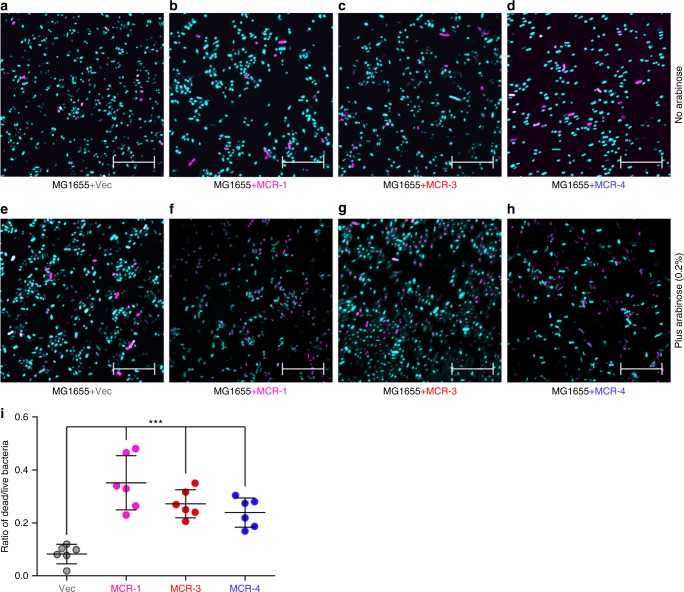
Effect on bacterial viability exerted by the expression of *mcr-1/3/4* genes. Bacterial viability measured without the addition of arabinose to *E. coli* MG1655 having the empty vector pBAD24 (**a**), MCR-1 (**b**), MCR-3 (**c**) or MCR-4 (**d**). Bacterial viability measured with the addition of 0.2% arabinose to the derivatives of *E. coli* MG1655 having the empty vector pBAD24 (**e**), MCR-1 (**f**), MCR-3 (**g**) or MCR-4 (**h**). The scale of bar is 20 μm. **i** The relative quantitation of LIVE/DEAD *E. coli* strains expressing MCR-1/3/4 0.2% (w/v) L-arabinose was supplemented to activate the expression of *mcr-1/3/4*. Bacterial cells were stained with LIVE/DEAD kit, giving the images with confocal laser scanning microscopy. Turquoise and magenta refers to live and dead cell. One-way analysis of variance (ANOVA) was applied, which is followed by Tukey–Kramer multiple comparisons post hoc test^36^. Statistical significance was fixed at *p* < 0.001

Very recently, our observations showed that expression of the *Neisseria* EptA, an intrinsic determinant of colistin resistance can more effectively repress the growth of its recipient host *E. coli* than mobilized colistin resistance determinants (MCR-1, MCR-2, and MCR-3) can^33^. However, the physiological effect of MCR-4 remains unclear. Here, we engineered a recombinant strain of *E. coli* MG1655 carrying pBAD24*::mcr-4* to address its potential effect on metabolic fitness (Supplementary Figure 11). First, the presence of 0.20% arabinose slightly inhibited bacterial growth of MG1655 alone (Supplementary Figure 11a) or with an empty vector pBAD24 (Supplementary Figure 11b), which is similar to the scenario with MCR-1 in TOPO 10^36^. As anticipated, an efficient expression of MCR-1 (Supplementary Figure 11c) and MCR-3 (Supplementary Figure 11d) does inhibit bacterial growth to some extent. However, it seems that MCR-4 does not inhibit as much as MCR-1 (or MCR-3) (Supplementary Figures 11d–e). Furthermore, we applied confocal microscopy to visually identify dead/alive *E. coli* cells with or without the expression of *mcr*-like genes (Fig. 6). In general similarity to that of the negative control and MG1655 with pBAD24 (Fig. 6a), no obvious increase in bacterial death of *E. coli* is seen in the absence of arabinose-triggered expression of *mcr-1* (Fig. 6b), *mcr-3* (Fig. 6c), or *mcr-4* (Fig. 6d). By contrast, the expression of either *mcr-1* (Fig. 6f), *mcr-3* (Fig. 6g), or *mcr-4* (Fig. 6h) gives a 4- to 5-fold increment in the relative percentage of cell death (Fig. 6i) when compared with that of the negative-control strain (Fig. 6e).

**Fig. 7:**
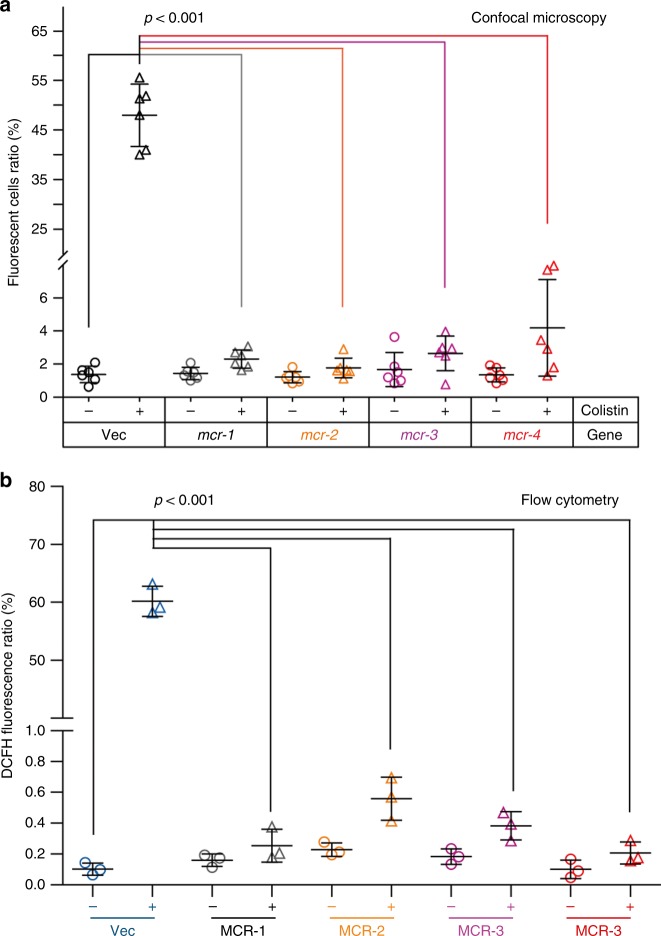
Relative quantification assays for colistin-induced ROS levels in *E. coli* expressing *mcr*-like genetic determinant. **a** Confocal microscopy-based quantification of ROS levels in *E. coli* carrying different versions of MCR family. **b** Use of flow cytometry to determine the ROS fluctuation in *E. coli* having *mcr* variants. The ratio of fluorescent cells (**a**) was calculated by counting the number of cells with/without fluorescence (seen in Supplementary Figure 10). Over 500 cells from 6 individual photographs were counted for each group. The data was given after one-way analysis of variance (ANOVA) along with Tukey–Kramer multiple comparisons post hoc test^36^. Statistical significance was set at *p* < 0.001. The flow cytometry data (**b**) was recorded with a BD FACSVerse flow cytometer counting 10,000 cells at a flow rate of 35 ml/min or 14 ml/min. DCFH florescence was excited with a 488 nm argon laser and emission was detected with the FL1 emission filter at 525 nm using FL1 photomultiplier tube

Colistin is known to primarily function by binding and disrupting the LPS layer of Gram-negative bacteria. In addition to this, colistin has also been shown to have an effect via an interaction with respiratory enzymes. This secondary mode of action is shown to be mediated by the production of reactive oxygen species (ROS) which can directly or indirectly disrupt Fe-S cluster-containing proteins, DNA and lipids (Supplementary Figure 12a). Given that MCR-4 can effectively modify lipid A in the LPS layer of bacteria (Supplementary Figure 12b), its downstream effect on ROS production was visualized by confocal microscopy in the presence of an oxidant-sensitive dye, DCFH2_DA (2′,7′-dichlorodihydrofluorescein diacetate) that produces intracellular fluorescence. In response to colistin exposure, fluorescence was observed in wildtype MG1655 cells (Supplementary Figure 13b). Confocal microscopy illustrated that ROS production was alleviated in cells expressing of *mcr-1*, *mcr-2*, *mcr-3,* or *mcr-4* during colistin exposure (Supplementary Figure 13d, f, h and j). Similar scenarios were also observed in flow cytometry-based detections (Supplementary Figure 14). FACS analyses showed that (i) the level of colistin-stimulated ROS level is dramatically boosts in the MG1655 without any *mcr*-like gene (Supplementary Figures 14a–b); (ii) the expression of *mcr-1* (Supplementary Figures 14c–d), *mcr-2* (Supplementary Figures 14e–f) and *mcr-3* (Supplementary Figures 14g–h), consistently quenches hydroxyl radical formation; and iii) colistin cannot activate the formation of ROS in the presence of *mcr-4* (Supplementary Figures 14i-j). Subsequently, the analyses of relative quantitation from both confocal microscopy (Fig. 7a) and flow cytometry (Fig. 7b) suggested that over 10- to 50-fold decrease in the total ROS level occurs in a certain strain of *E. coli* expressing a given version of MCR family ranging from *mcr-1* to *mcr-4*.

Since hydroxyl radicals produced by the Fenton reaction are thought to be a major contributor to ROS mediated cell death, we sought to utilize a ferric chelator, bipyridine to rescue them. Addition of bipyridine increases cellular survival of bacterial cells of *E. coli* at a level comparable to the effect of expressing *mcr-1* (Supplementary Figure 12c). A similar scenario is observed when a scavenger L-cysteine is used (with/without bipyridine). Given the fact i) that the expression of *mcr-4* has the same effect on cell survival as bipyridine or L-cysteine (Supplementary Figure 12c) and ii) that it abolishes ROS production in the presence of colistin (Fig. 7 and Supplementary Figure 13j), we believe that the modification of lipid A by MCR-4 prevents colistin entry into the cell and hence prevents any of its downstream effects mediated through ROS production.

## Discussion

Resistance to colistin has become a global challenge with a remarkable number of new members being detected and added to the MCR family. The recently-identified *mcr-4* and a number of its variants have been predominantly discovered in *E. coli* or *Salmonella enterica* isolates from food-producing animals and humans. Such *mcr* genes mobilized on plasmids to clinical pathogens are the leading cause hospital-borne infections worldwide^37^. In structural similarity to the integral enzyme MCR-1, MCR-4 also contains a N-terminal transmembrane region connected by a flexible linker with C-terminal catalytic domain (Supplementary Figures 3a–b). Surprisingly, MCR-4 is more closely related to the intrinsic resistance determinant EptA, encoded on the chromosome of *Neisseria* than to the mobilized MCR-1 family and this study shows that MCR-4 forms a phylogenetic group distinct from MCR-1. This is further echoed by the fact that the two domains of MCR-4 and MCR-1 are not functionally interchangeable. However, unlike EptA, both MCR-4 and MCR-1 impart a 4- to 8-fold greater resistance to colistin (16 μg/ml) to a susceptible *E. coli* host. This suggests a level of functional similarity between the two despite their evolutionary and overall structural differences. In fact, the entire diverse family of MCR and ICR enzymes seem to share a similar active site organization^38^ and a ping-pong mechanism of catalysis. It has been confirmed both in vivo and in vitro that MCR-4 can transfer a PE moiety from a PE lipid donor to 4’-position of LPS-lipid A in a two-step reaction involving an enzyme-bound PEA intermediate, a mechanism shared by the entire MCR family (Supplementary Figure 3c). This is distinct from other physical bases for colistin resistance which include either modification of the LPS-lipid A with an addition of the cationic sugar 4-amino-4-deoxy-l-arabinose^39^ or attachment of a glycine to glucosamine residues via a tripartite system^16^. The resulting modification of lipid A by MCR-4 also alleviates ROS production by wild-type *E. coli* in response to colistin. As a secondary target, the type II NADH-quinone oxidoreductase (NAD-2, an inner membrane enzyme) is inhibited by colistin in both Gram-positive and Gram-negative bacteria^40^. Of note, bacterial killing by colistin is associated with a hydroxyl radical death pathway^41^. We show that the addition of iron chelators and scavengers can also negate the effect of colistin and aid in cell survival. This suggests the involvement of the Fenton reaction and production of free hydroxyl radicals that are known to attack DNA, proteins, and lipids^42^.

Another recently-discovered gene *mcr-3* also behaves in a manner similar to *mcr-4*. MCR-3 is, however, quite different from MCR-1 and MCR-2 and forms a separate subclade from MCR-4. Despite being phylogenetically-closer to MCR-4 than MCR-1, domain-swapping experiments confirm that the two domains of MCR-3 and MCR-4 are incompatible. Interestingly though, MCR-3 is weaker than both MCR-1 and MCR-4 in terms of colistin resistance levels (~8 μg/ml). The source of the genetic diversity is puzzling and is a has been the source of much interest. The broad division of the MCR family into three subclades suggests an evolutionary divergence from a common ancestor (Fig. 1a). Interestingly, all three groups contain mobilized as well as intrinsic *mcr* genes (Fig. 1a). In fact, the intrinsic resistance determinant EptA is more closely-related to Subclade II harboring MCR-3, while others like ICR-Mo and related proteins from *Moraxella* are phylogenetically-closer to the MCR-1/2-containing Subclade I (Fig. 1a). Along with several related members exclusively present in *Shewanella* (Fig. 1b), MCR-4 variants are grouped into Subclade III (Fig. 1a). It seems likely that these groups are evolving independently, and that their current source of genetic diversity is held in chromosomal reservoirs. For MCR-1/2, it has been suggested that *Moraxella*, which are routinely found along with member of *Enterobacteriaceae* in swine gut microbial populations, might act as a chromosomal reservoir for genetic diversity in the MCR-1/2 family. Interestingly, we observe that the mobilized versions of MCR-3 and MCR-4 found in gut bacteria show very high homology to genes on the chromosomes of *Aeromonas* and *Shewanella* species^23^, respectively. These are predominantly marine microorganisms and are known to be fish commensals/pathogens. This might represent an important reservoir for providing genetic diversity to the extant MCR-3/4 genes mobilized in clinical strains. In fact, studies from China and Vietnam^43,44^ have also suggested a zoonotic mode of transmission of *mcr*-like genes. Given that colistin has been extensively and indiscriminately used as a feed supplement in both animal husbandry and aquaculture, this is completely plausible.

The functional unification of the MCR family, despite their evolutionary differences, is critical to developing future therapies. Current scientific awareness has led to policy changes around the world regarding both colistin use as well as surveillance for colistin resistance. The number of variants being identified is alarming. Since colistin is one of the few ‘last-resort’ therapeutic options, it is important to get a complete mechanistic understanding of the entire family of colistin resistance determinants. Our findings provide a complete functional characterization of MCR-4 while putting it in an evolutionary context with the growing body of MCR-like genes. This might provide mechanistic basis for developing better strategies to mitigate colistin resistance from adjuvants to antibiotic modifications.

## Methods

### Strains, plasmids, and growth conditions

All the *E. coli* strains are listed in Supplementary Table 1, while DNA oligo sequences are collected in Supplementary Table 2. To produce modest amounts of membrane protein MCR-4, an arabinose-inducible vector pBAD24 was modified via introduction of an eight histidine (8× his) tag at the 3’-end of the multiple cloning site, giving the final version, pBAD24.8 × his (Supplementary Table 1)^29,33^. In total, three types of *E. coli* strains were utilized here, namely MG1655, DH5α, and Transetta DE3 (Supplementary Table 1). MG1655 is utilized as a recipient of *mcr-4* (and/or its mutants) to test bacterial viability in the presence of colistin, *E. coli* DH5α is a cloning host and *E. coli* Transetta DE3 is the protein expression host (Supplementary Table 1)^45,46^. Bacterial growth was examined at 37 °C with liquid Luria-Bertani (LB) broths or LB agar plates^10,45,46^.

### Genetic manipulations

*mcr-4* was synthesized in vitro by polymerase chain assembly as described for *mcr-2*^35^. Domain-swapped versions between *mcr-4* and *mcr-1* (and/or *mcr-3*) were produced by overlap polymerase chain reaction (Supplementary Table 2). The mosaic versions produced referred to TM1-MCR-4, TM4-MCR-1, TM3-MCR-4, and TM4-MCR-3, respectively (Supplementary Table 1). Site-directed mutagenesis was utilized to give an array of point-mutants of *mcr-4* as reported by Xu et al.^9,10^. The aforementioned PCR system was established using the Mut Express II fast mutagenesis kit V2 (Vazyme Biotech Co., Ltd.) with series of specific sets of primers (Supplementary Table 2). All the resultant plasmid constructs were validated by multiplex PCR as well as by direct DNA sequencing.

### Determination of colistin resistance

Bacterial viability of *E. coli* alone or with *mcr-4* (and/or its derivatives) were recorded in LBA plates with colistin^47,48^. 0.2% arabinose was supplemented into LBA plates to induce functional expression of MCR-4 and the derivatives. As recommended by European Committee on Antimicrobial Susceptibility Testing (EUCAST), the minimum inhibitory concentration (MIC) of colistin was calculated with Cation-Adjusted Mueller-Hinton Broth CAMHB^46,49^. A fresh culture in CAMHB medium whose optical density at the wavelength of 600 nm (OD600) was around 0.5, was diluted 100-fold in CAMHB with different levels of colistin (ranging from 0 to 0.25, 0.5, 1.0, 2.0, 4.0, 8.0, 16.0, and 32.0 μg/ml) and incubated in a shaker (220 rpm, at 37 °C) for 16 h^10,45,46^.

### Expression and purification of MCR-4

Unlike recently-described plasmid-borne MCR-1/2^9,10,29,35^ and the two chromosomally-encoded intrinsic colistin resistance determinants [Neisserial EptA^9,45,46^ and ICR-Mo^29^], MCR-4 is degraded when the BL21(DE3)/pET21a [and/or pET28a] system is utilized. To avoid this, an improved pBAD24 vector containing a carboxy-terminal octa-histidine tag (pBAD24.8 × His) was developed and utilized (Supplementary Table 1). Efficient expression of MCR-4 membrane protein was triggered through the addition of arabinose (0.1%) into the mid-log phase culture of *E. coli* Transetta DE3 cells carrying pBAD24.8 × his^29,33^.

As described with MCR-1/2^9,10,35,46^, bacterial cultures were harvested by centrifugation, re-suspended in Buffer A [20 mM Tris-HCl (pH 8.0), 50 mM NaCl, 5 mM DNase I, 1 mM phenylmethylsulfonyl fluoride and 2 mM MgCl_2_], and lysed by three rounds of passages (i.e., once at 500 p.s.i. and twice at 1300 p.s.i.) through a French press (JN-Mini, Guangzhou, China). First, the resultant lysates were subjected to two rounds of centrifugation (i.e., 1 h of spinning at 16,800 rpm at 4 °C for^9,35^ followed by 1 h of centrifugation at 38,000 rpm at 4 °C), with MCR-4 found in the pellet. Second, the pellet was solubilized with Buffer B [20 mM Tris-HCl (pH 8.0), 50 mM NaCl, 5 mM imidazole, 5% glycerol and 1% detergent DDM (m/v)] and kept overnight at 4 °C. Third, the clarified supernatant of the overnight mixture was incubated with pre-equilibrated Ni-NTA agarose beads for 4 h at 4 °C^9,10^. Finally, MCR-4 was eluted with elution buffer [20 mM Tris-HCl (pH 8.0), 50 mM NaCl, 200 mM imidazole, 5% glycerol (v/v), 0.03% DDM(m/v)] after the removal of contaminants with wash buffer [20 mM Tris-HCl (pH 8.0), 50 mM NaCl, 10–40 mM imidazole, 5% glycerol (v/v), and 0.03% DDM (m/v)]^9,10,29,35^. The purified MCR-4 protein was further concentrated with a 30-kDa cut off ultra-filtration column (Millipore), The protein purity was judged by SDS-PAGE (12%), and its identity was verified using a Waters Q-Tof API-US Quad-ToF mass spectrometer^50,51^.

### Biophysical characterization of MCR-4

To further characterize the biophysical properties of MCR-4, two different technologies were adopted, namely circular dichroism (CD) and inductively coupled plasma mass spectrometry (ICP-MS). CD was used to probe the secondary structure of MCR-4^9,10,29^ and the CD spectra was recorded on a Jasco model J-1500 spectrometer (Jasco Corp., Tokyo, Japan) by continuous wavelength scanning (in triplicate) from 200 to 260 nm at a scan rate of 50 nm/min^52^ and smoothed with a Savitsky-Golay filter^53^. ICP-MS was utilized to examine the presence of zinc within the MCR-4 protein^9,10,29^. In brief, MCR-4 protein (~0.2 mg/ml) was loaded on to a NexION 300TM ICP-MS instrument (PerkinElmer Life Sciences) switched to Collision-Cell mode. Mass-to-charge ratio (m/z) was measured using kinetic energy discrimination mode with helium as the carrier gas^54^.

### Structural determination of LPS-lipid A

A pool of crude lipopolysaccharide (LPS)-lipid A species were extracted from *E. coli* with or without any version of *mcr*-like genes using the method of Liu and coworkers^8,55^ with minor changes. This crude samples were treated with DNase I (25 ug/ml) and RNase A (100 ug/ml) to remove contaminating nucleic acids and then with Proteinase K for 1 h to remove protein contaminants^10^. The Kdo linkage was then cleaved in a 10 mM sodium acetate buffer (pH 4.5) with aqueous 0.2% SDS at 100 °C for 1 h to give purified lipid A. Residual SDS was progressively cleaned by precipitating with acidified ethanol^56^ followed by two rounds of washing with 100 μl of 95% ethanol, and a final round of wash with 1 ml ethanol^57^.

The purity of lipid A was confirmed by SDS-PAGE^58^ followed by silver staining. The chemical structure of lipid A was determined by MALDI TOF/TOF mass spectrometry (Bruker, ultrafle Xtreme) in negative-ion mode with the linear detector^16,59^. In general, the qualified lipid A species were dissolved in 20 ul of chloroform/methanol solution and then mixed with 2.5-dihydroxybenzoic acid matrix chloroform/methanol/water (3:1.5:0.25) (20 mg/ml) solution at a ratio of 1:1. 1ul of the resulting lipid A solution was loaded onto a MALDI sample plate, giving a unique MS spectrum^9^. Indeed, every spectrum in our trials was generated from an average of 500 shots and 50% laser power^9,10,29^.

### In vitro enzymatic assay for MCR-4 action

As initially described by Anandan et al.^60^, an enzymatic reaction was established to test MCR-4 action. The fluorescent substrate 1-acyl-2-{12-[(7-nitro-2-1,3-benzoxadiazol-4-yl) amino] dodecanoyl}-sn-glycero-3-phosphoethanolamine (Avanti Lipids, USA) abbreviated as NBD-glycerol-3-PEA was used to test MCR-4. The in vitro system [50 μl in total, 50 mM HEPES (pH 7.5), 100 mM NaCl, 0.03% of DDM, 0.2 mM NBD-glycerol-3-PEA, and 30 μM MCR-4] was kept at 25 °C for around 20 h. Similar to MCR-1/2^9,10^ and ICR-Mo^29^, thin layer chromatography (TLC) was also utilized to separate the NBD-glycerol product from the MCR-4 reaction mixture in a mobile phase [ethyl acetate: methanol: water, 7:2:1 (vol/vol)]^9,10,29^. The NBD-glycerol product was distinguished from the substrate NBD-glycerol-3-PEA by exposing the TLC plate to Epi blue light (455–485 nm) and visualizing the fluorescent signals with a gel imaging system (Bio-Rad)^60^. Using NBD-glycerol-3-PEA, the MCR-4 reaction mixture was further subjected to chemical identification by liquid chromatography mass spectrometry (Agilent technologies 6460 Triple Quad LC/MS)^61^. Using Zorbax SB C18 (2.1*50 mm, 3.5 μm) as analytical chromatographic column, the aforementioned samples were eluted with methanol/0.1% methanoic acid (95:5) at 0.3 ml/min., as utilized recently^9,10,29^.

### Measurement of physiological function of MCR-4 in *E. coli*

To address bacterial fitness cost caused by *mcr*-4 and other *mcr*-like genetic determinants, we measured growth curves of MG1655 strains (Supplementary Table 1) and performed bacterial “LIVE/DEAD” staining assays. In the latter, the images were captured with a Confocal Laser Scanning Microscopy (CLSM, Zeiss LSM 800) as recently described^29,33^.

To probe the effect of MCR expression on the de novo formation of hydroxyl radicals, we carried out the experiment of chemical rescue. Two ROS inhibitors involved in this assay refer to bipyridine (the ferric chelator) and L-cysteine (a ROS scavenger), respectively^62,63^. The intracellular accumulation of ROS was monitored through the staining with an oxidant sensor dye DCFH2-DA (i.e., 2′,7′-dichlorodihydrofluorescein diacetate, Sigma). Subsequently, both confocal microscopy^29^ and flow cytometry (BD FACSVerse flow cytometer)^64^ were applied to visualize the ROS production and quantify the relative level of intracellular ROS.

### Flow cytometry-based measurement of the intracellular ROS level

A mid-log phase culture of *E. coli* (OD600, 0.5) was utilized to measure the intracellular level of ROS, in which 10 mM of oxidant sensor dye DCFH2-DA (Sigma) was supplemented and maintained for 0.5 h. When necessary, colistin was added. Subsequently, bacterial samples were diluted with PBS to 10^6^ CFU/ml prior to the flow cytometry assays^65^. As a result, FACS data was recorded with a BD FACSVerse flow cytometer through counting 10,000 cells at a flow rate of 35 ml/min (and/or 14 ml/min). DCFH florescence was excited with a 488 nm argon laser and emission was detected with the FL1 emission filter at 525 nm using FL1 photomultiplier tub. No less than three independent experiments were conducted.

### Structure modeling and molecular docking

The structure of full-length MCR-4 was modeled using Swiss-Model (https://swissmodel.expasy.org/interactive/qMEvX5/models/)^66^ and EptA from *Neisseria meningitidis* [PDB: 5FGN]^60^ as a template. An appreciable coverage score along with the decent QMEAN value (that provides a global and local absolute quality estimate on the modeled structure^67^) verified the suitability of the structural prediction.

Molecular docking was performed using the UCSF DOCK 6.7 software (version 6.7)^68^ to study binding of MCR-4 enzyme to its phosphatidylethanolamine (PE) lipid substrate. The ready-to-dock 3D structure of phosphatidylethanolamine (ID: ZINC32837871) and its head group (ID: ZINC02798545) was acquired from the ZINC database^69^. Protein structure was optimized for molecular docking using UCSF Chimera software^70^. Favorable orientation of PE in MCR-4 was searched in a 20 Å space around complexed ligand dodecyl-β-D-maltoside (DDM) in EptA (PDB: 5FGN). Given that the modeled architecture of MCR-4 appears in a compact (and even closed) state during its dynamics, and thus cannot provide enough cavity space to hold the full PE molecule with flexible acyl chains, only head group of PE molecule was utilized for docking into MCR-4 structure. The LigPlot+ software was run to give the diagram for two-dimension ligand-protein interaction^71^. Alternatively, because the PE lipid substrate is well docked into EptA, the putative model of MCR-4 docking with full-length of PE was obtained by structural superposition of MCR-4 with EptA.

### Phylogenetic analysis

A protein BLAST with standard options was performed with the amino acid sequence of MCR-4 used as a query. Models and uncultured environmental samples were excluded and the BLASTP parameters were modified to display 500 target sequences. All matches with greater than 70% query coverage and at least 30% identity were selected and exported. MCR-1, MCR-2 and ICR-Mo and its homologs were manually included. Unique sequences were identified using the Uniqueseq server (https://www.ncbi.nlm.nih.gov/CBBresearch/Spouge/html_ncbi/html/fasta/uniqueseq.cgi) before performing a multiple sequence alignment using MUSCLE (https://www.ebi.ac.uk/Tools/msa/muscle/). A total of 51 unique amino acid sequences (or 16 sequences for the subset tree) were utilized for the subsequent phylogenetic analysis. jModeltest (via MEGA 7^72^ was used to identify the best-fit amino acid substitution model and the best model was used to generate a maximum-likelihood tree with 1000 bootstrap replicates.

Initial tree(s) for the heuristic search were obtained automatically by applying Neighbor-Joining and BioNJ^73^ algorithms to a matrix of pairwise distances estimated using the JTT approach, and then selecting the topology with superior log likelihood value. A LG model^74^ with a discrete Gamma distribution and Invariant sites was used to model evolutionary rate differences among sites [5 categories (+G, parameter = 0.7355)].

## Supplementary information

Supplementary information

## Data Availability

All the data of this study are included in the main text and supplementary information files. Any additional source data or material used in this study can be obtained from the corresponding author upon reasonable request.
