# Supplementary material for: Action and mechanism of the colistin resistance enzyme MCR-4
Source: Commun Biol. 2019 Jan 25;2:36. doi: 10.1038/s42003-018-0278-1 (PMC6347640; doi:10.1038/s42003-018-0278-1)
Supplement: Supplementary file 1 — Supplementary information [file 42003_2018_278_MOESM1_ESM.pdf]

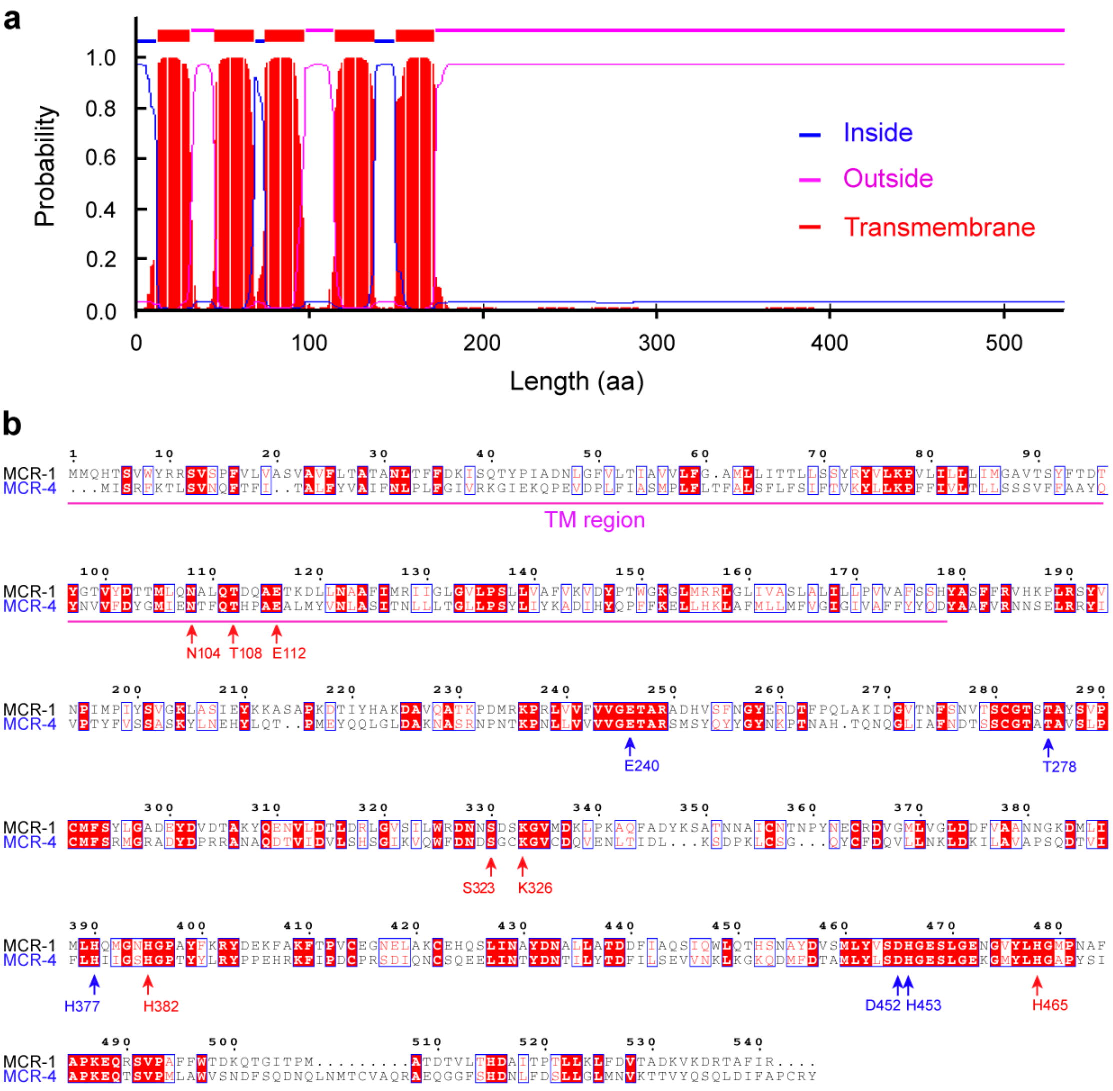

Supplementary Figure 1 Bioinformatic analyses of MCR-4

a. Prediction of the transmembrane region of MCR-4 protein

b. Protein sequence alignment of MCR-4 with MCR-1

The zinc-binding sites are indicated with blue arrows, whereas the putative PE-interactive residues are highlighted with red arrows.

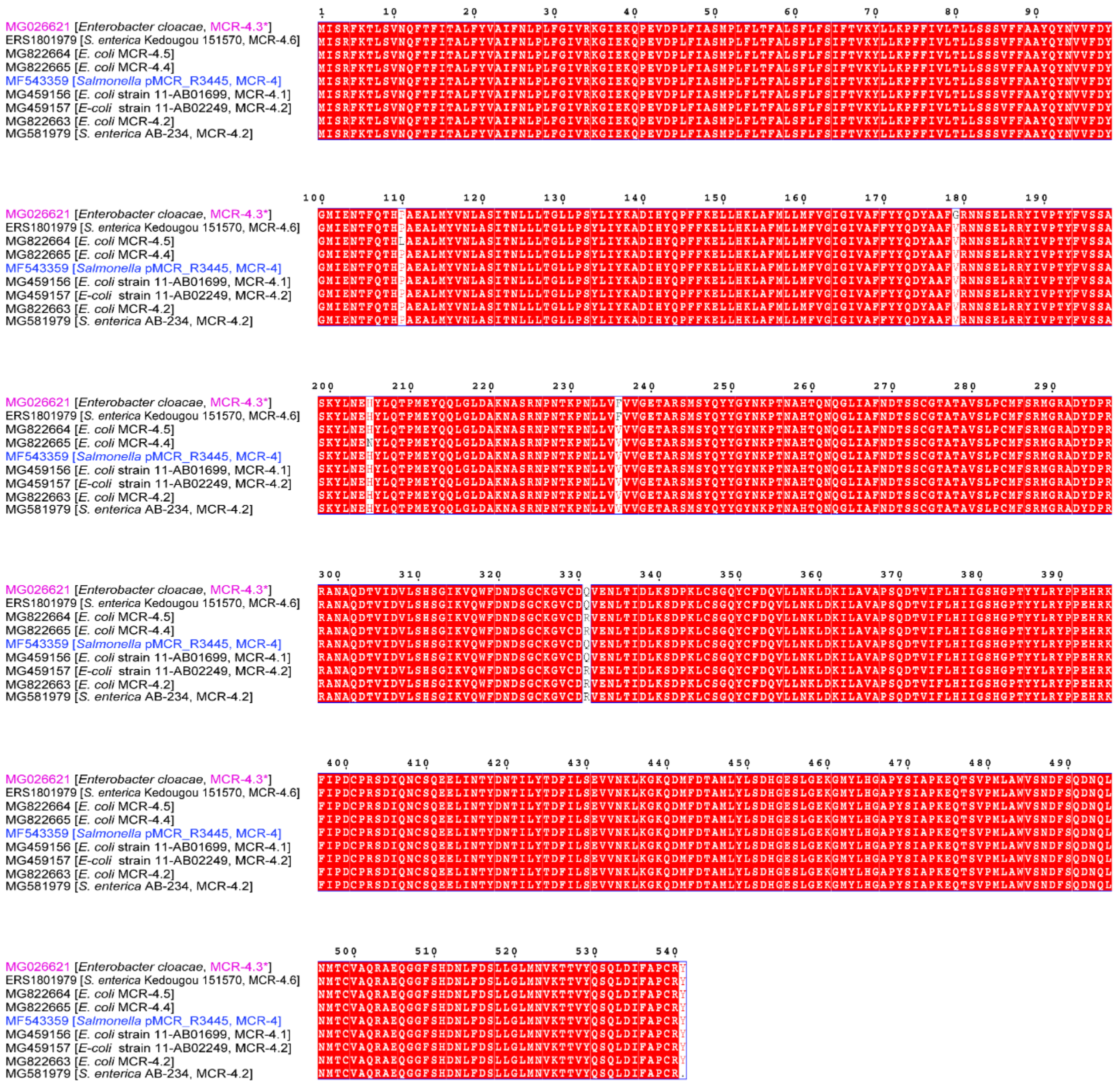

Supplementary Figure 2 Multiple sequence alignments of MCR-4 and its variants

* *mcr-4.3* renamed from a redundant *mcr-4.2* deposited into GenBank database, is an inactive variant of *mcr-4* with only two single mutations (V179G and V236F) [1].

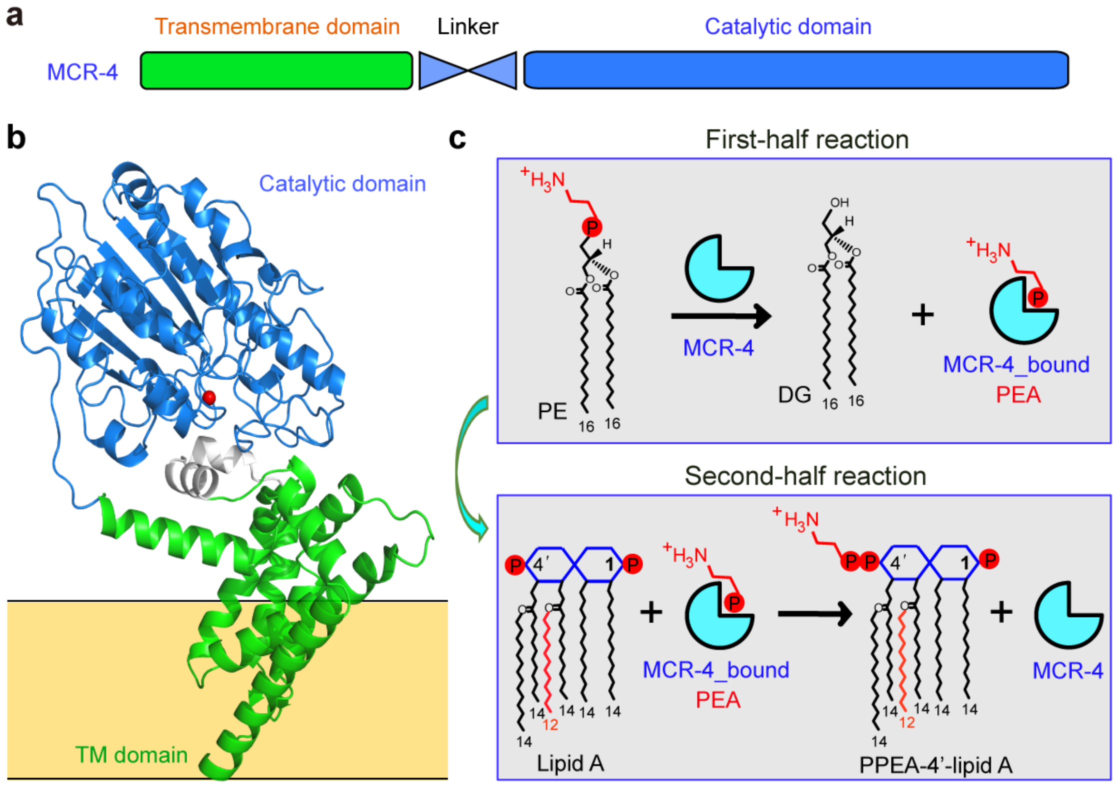

Supplementary Figure 3 A working model proposed for MCR-4 colistin resistance

a. Cartoon illustration of functional motifs in MCR-4

b. Ribbon presentation of modeled structure of full-length MCR-4

c. Scheme for MCR-4 action in catalyzing the transfer of PER from PE to lipid A

Linker (**panel a**) indicates a region containing a flexible loop between the TM domain and catalytic domain [2]. MCR-4-bound PEA (**panel c**) denotes an adduct of the enzyme MCR-4 complexed with PEA [2]. As described with MCR-1/2/3 [3-5], structural modeling was conducted via SWISS-MODEL and the ribbon structure was generated with PyMol. The structural template refers to the neisserial EptA with known structure (PDB: 5FGN) [6]. The ping-pong enzymatic model for MCR-4 action (**panel c**) is modified appropriately from those recently assigned to MCR-1/2 [2, 4, 5, 7] with permissions.

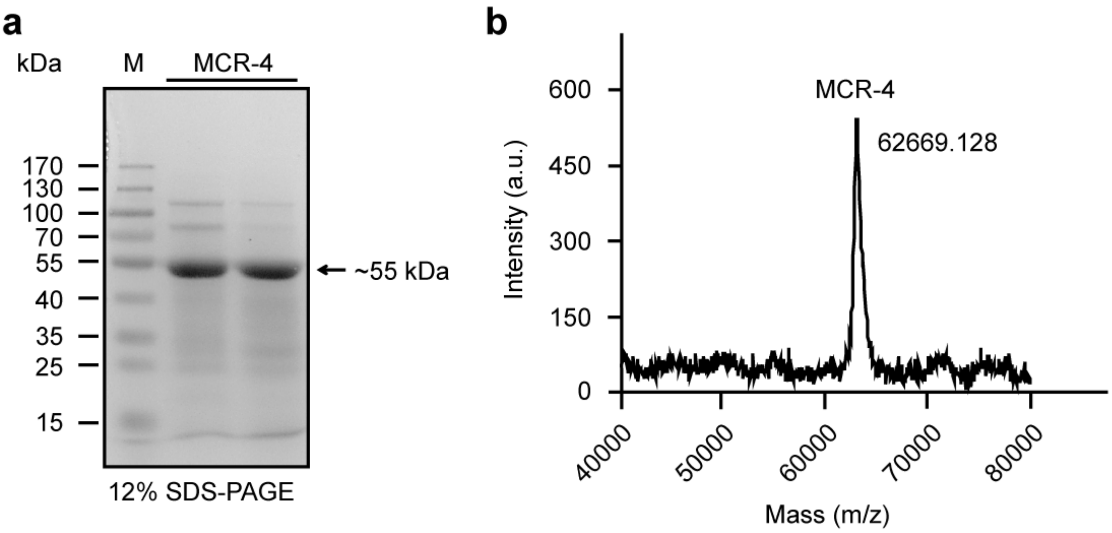

c

1 **MISRFKTLSV NQFTFITALF YVAIFNLPLF GIVRKGIEK**Q PEVDPLFIAS MPLFLTFALS

61 FLFSIFTVKY LLKPFFIVLT LLSSSVFFAA YQYNVVFDYG MIENTFQTHP AEALMYVNLA

121 SITNLLLTGL LPSYLIYK**AD IHYQPFFKEL LHKLAFMLLM FVGIGIVAFF YYQDYAAFVR**

181 NNSELRR**YIV PTYFVSSASK YLNEHYLQTP MEYQQLGLDA K**NASR**NPNTK PNLLVVVVGE**

241 **TAR**SMSYQYY GYNKPTNAHT QNQGLIAFND TSSCGTATAV SLPCMFSRMG RADYDPR**RAN**

301 **AQDTVIDVLS HSGIKVQWFD NDSGCKGVCD QVENLTIDLK** SDPK**LCSGQY CFDQVLLNK**L

361 **DKILAVAPSQ DTVIFLHIIG SHGPTYYLR**Y PPEHRKFIPD CPR**SDIQNCS QEELINTYDN**

421 **TILYTDFILS EVVNK**LK**GKQ DMFDTAMLYL SDHGESLGEK GMYLHGAPYS IAPKEQTSVP**

481 **MLAWVSNDFS QDNQLNMTCV AQRAEQGGFS HDNLFDSLLG LMNVKTTVYQ SQLDIFAPCR**

541 Y

Coverage: 70.31%

Supplementary Figure S4 Preparation and identification of the recombinant MCR-4 membrane protein

a. SDS-PAGE (15%) profile of the purified MCR-4 protein

b. Use of MALDI-TOF to determine molecular mass of the recombinant integral membrane protein of MCR-4

c. MS identity of MCR-4

The peptides matched to MCR-4 are shown in underlined/bold letters. MS result suggests that the digested peptide fragment exhibit 70.31% coverage to that of MCR-4.

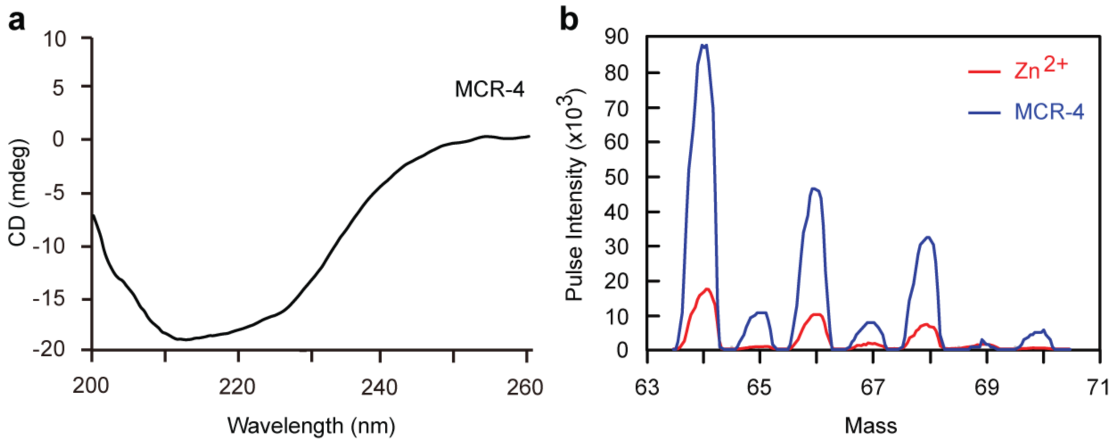

Supplementary Figure 5 Biophysical properties of MCR-4 protein

a. Circular dichroism (CD) spectrum of MCR-4 protein

The CD result indicates that the secondary structure of MCR-4 protein has high percentage of α-helices since it has a typical spectrum at 210-220 nm

b. Use of inductively-coupled plasma mass spectrometry (ICP/MS) to probe the presence of zinc ions in MCR-4 protein

ICP/MS result suggests that MCR-4 is a zinc-bound protein. Zn^{2+} is used here as positive control.

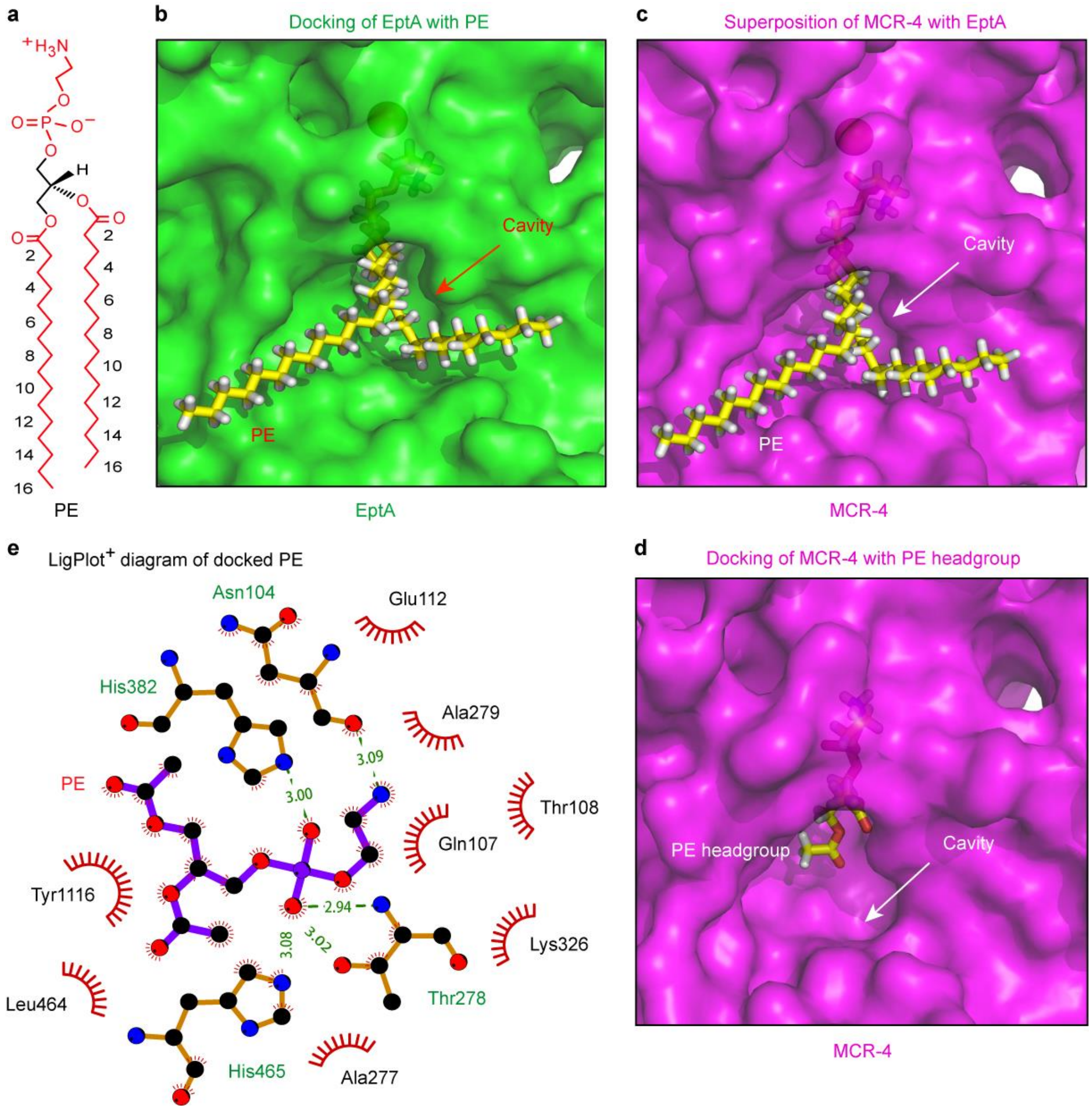

Supplementary Figure 6 Structural illustration of PE cavity in MCR-4

a. Chemical structure of PE

b. Snapshot of a PE cavity in EptA revealed by its docking with PE

c. A putative PE cavity of MCR-4 suggested by structural superposition

d. An enlarged view of a PE cavity revealed by docking of MCR-4 with PE headgroup

e. Ligplot^{+} diagram of PE headgroup docked into MCR-4

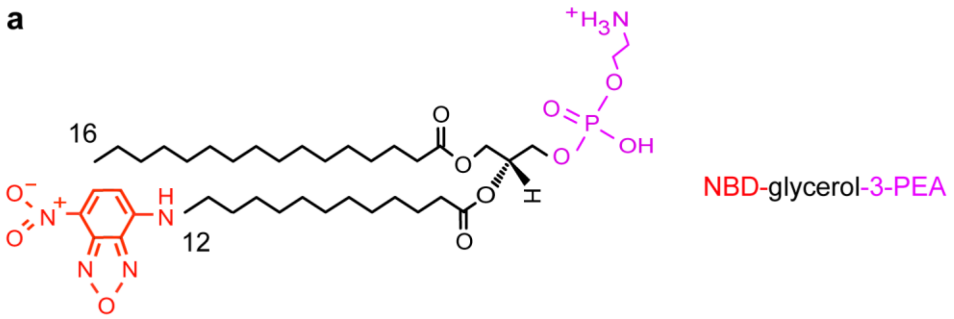

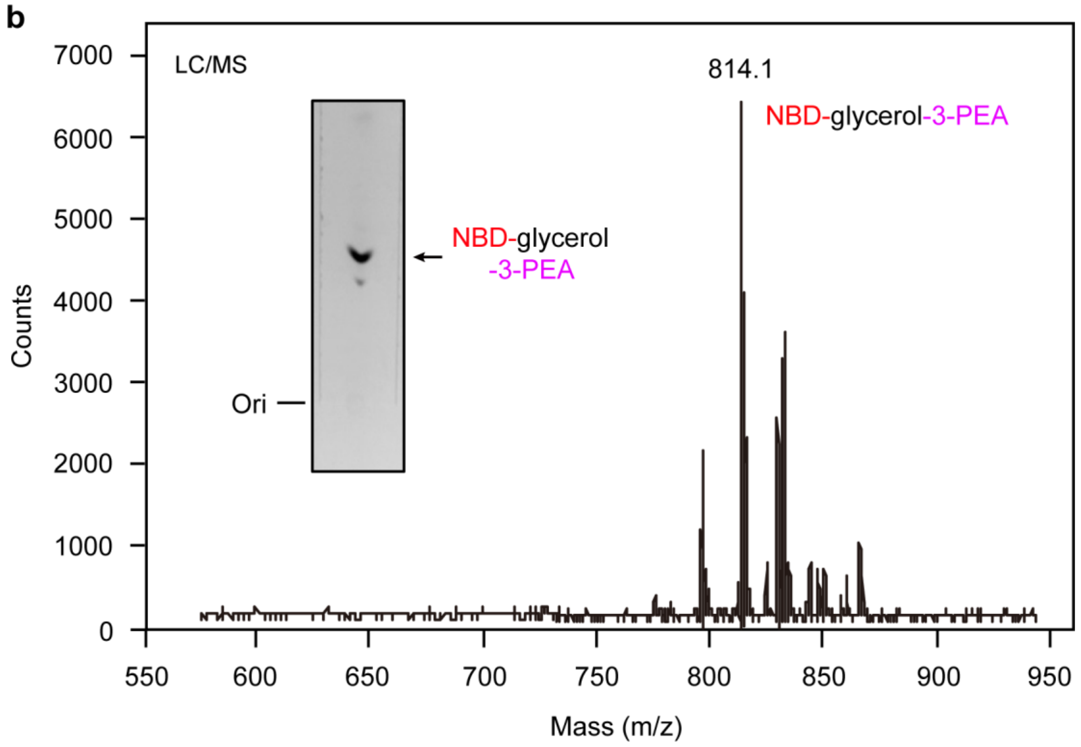

Supplementary Figure 7 Identity of NBD-glycerol-3-PEA, an alternative substrate of PE

a. Chemical structure of NBD-glycerol-3-PEA

b. LC/MS verification of NBD-glycerol-3-PEA

The inlaid gel denotes the TLC profile of NBD-glycerol-3-PEA.

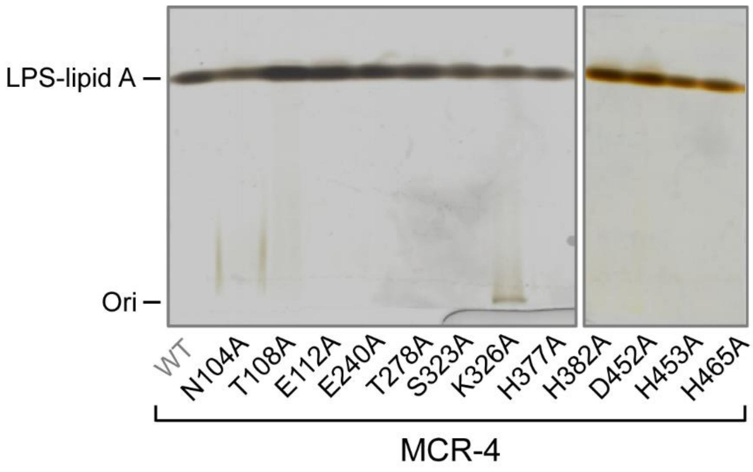

Supplementary Figure 8 Silver-staining analysis of the lipopolysaccharide (LPS)-lipid A pools from *E. coli* with/without expression of *mcr-4* (and/or its derivatives)

SDS-PAGE (12%) is used to separate the different samples of LPS-lipid A (electrophoresis parameters: 1mA, 3-4 hrs). Two independent gels were combined together to give the above photograph because the limited well numbers of each gel (10 wells per gel).

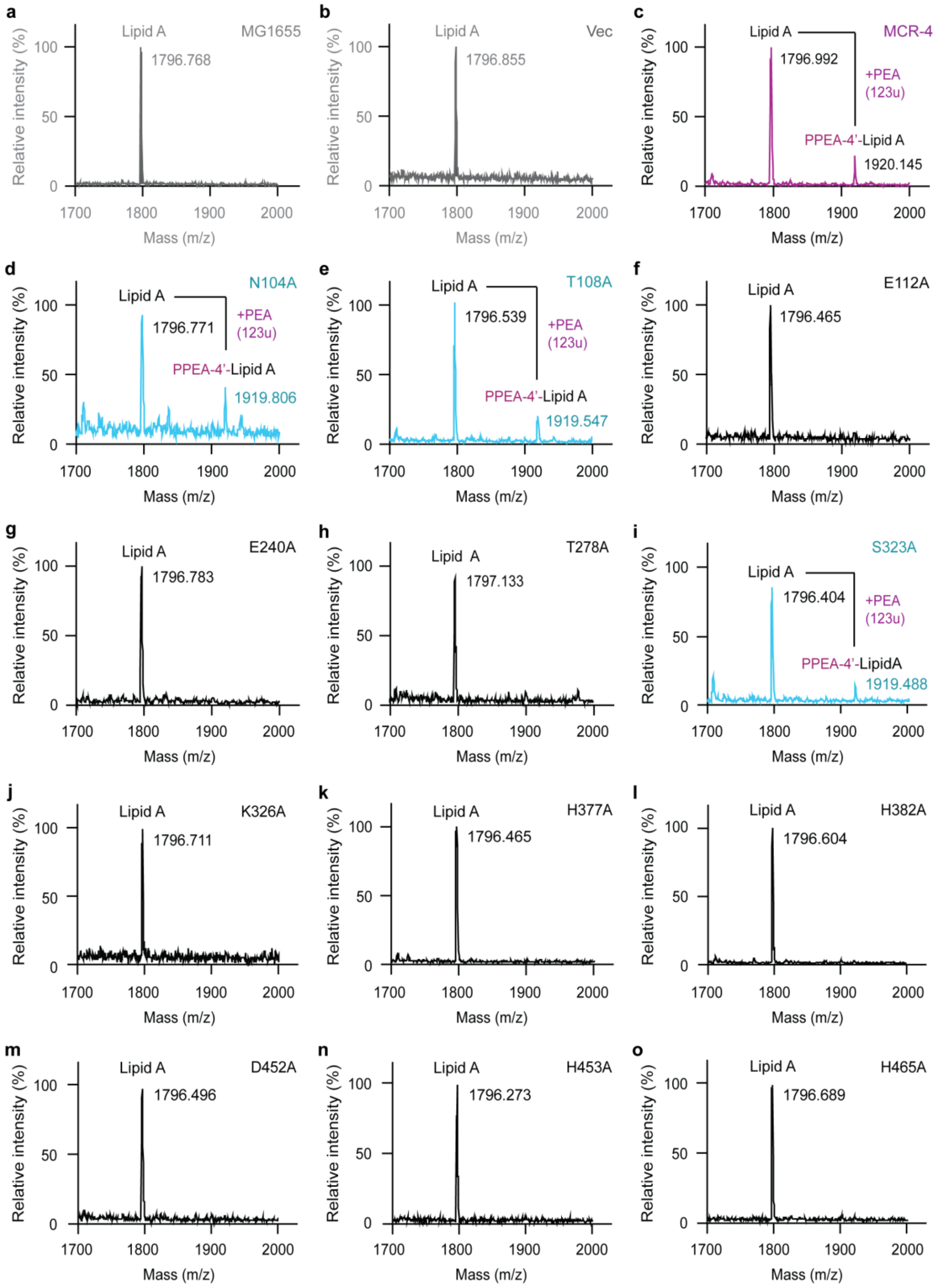

Supplementary Figure 9 MALDI TOF mass spectra elucidating the physiological roles of the PE lipid substrate-interactive cavity of MCR-4 in chemical modification of the lipid A moieties of lipopolysaccharides in *E. coli*

a. MS spectrum of the LPS-lipid A species isolated from the colistin-susceptible strain *E. coli* MG1655 alone

b. MS profile of the LPS-lipid A species of the *E. coli* MG1655 strain containing the empty vector pBAD24
c. MCR-4 modifies lipid A (m/z, 1796.992), giving the PPEA-4'-lipid A (m/z, 1920.145)

The point-mutants N104A (**d**), T108A (**e**) and S323A (**i**) in MCR-4 retained partial activity in catalyzing the chemical modification of lipid A moieties.

The three point-mutants of MCR-4 [namely E112A (**f**), E240A (**g**) and T278A (**h**)] are inactive in the transferring of PEA to suggestive 4'-phosphate position of lipid A species.

None of the following six point-mutants of MCR-4 [namely, K326A (**j**), H377A (**k**), H382A (**l**), D452A (**m**), H453A (**n**), and H465A (**o**)] possess enzymatic activity.

Of note, the MS peak of lipid A species appears at m/z of 1796.404~1797.133. Expression of functional (and/or partial active) versions of *mcr-4* in *E. coli* results in the presence of its modified form PPEA-4'-lipid A at m/z of 1919.488~1920.145.

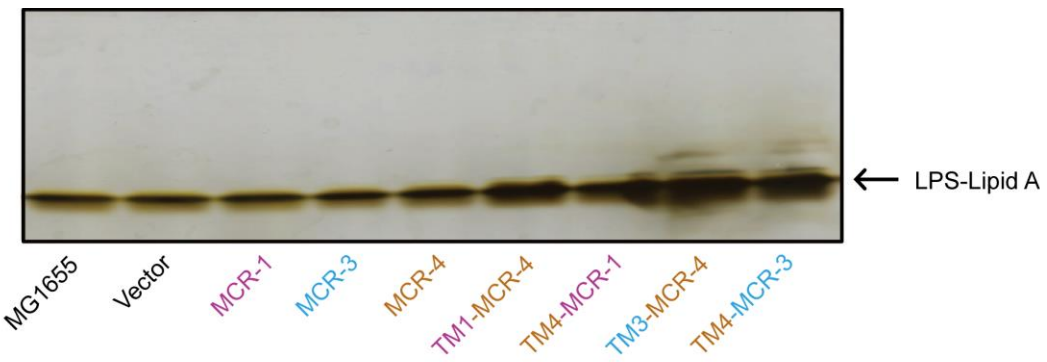

Supplementary Figure 10 Silver-staining analysis of the LPS-lipid A species isolated from *E. coli* expressing different domain-swapped versions of *mcr-4* and *mcr-1/3*

The separation of LPS-lipid A was performed as described in **Supplementary Figure 7**.

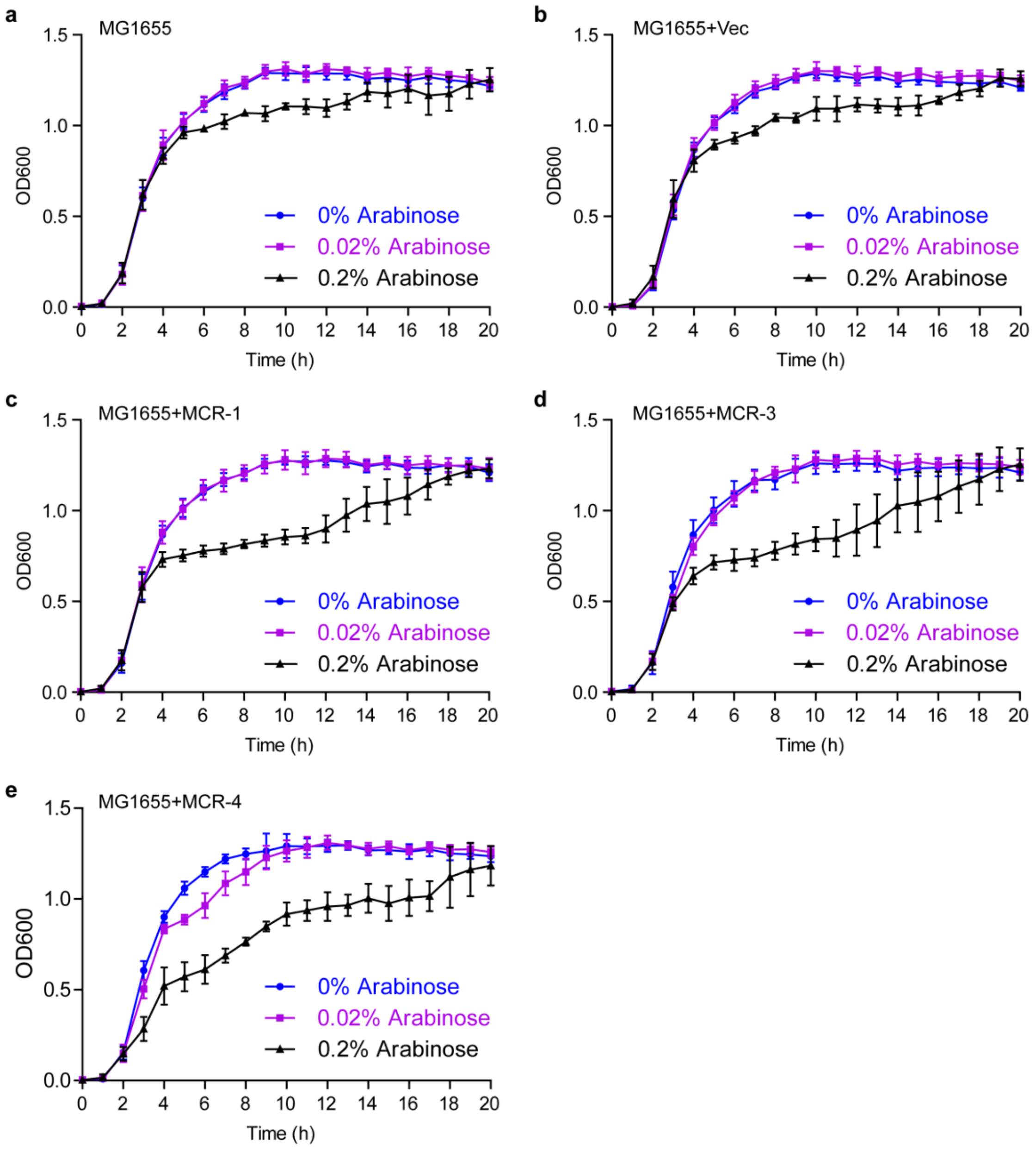

Supplementary Figure 11 Bacterial growth is slightly interfered by the family of MCR proteins

Bacterial growth curves of the two-negative control strains, *E. coli* MG1655 alone (**a**) and *E. coli* MG1655 carrying the empty vector pBAD24 (**b**)

Growth curves of *E. coli* MG1655 expressing MCR-1 (**c**), MCR-3 (**d**) or MCR-4 (**e**)
The expression of *mcr-1* and/or (*mcr-3* and *mcr-4*) was induced by the addition of varied levels of arabinose (0.00%, 0.02%, and 0.20%) into LB media. Three independent experiments were performed here. It is expressed with means ± SD. The value of OD600 was recorded in triplicate. SD denotes standard deviation.

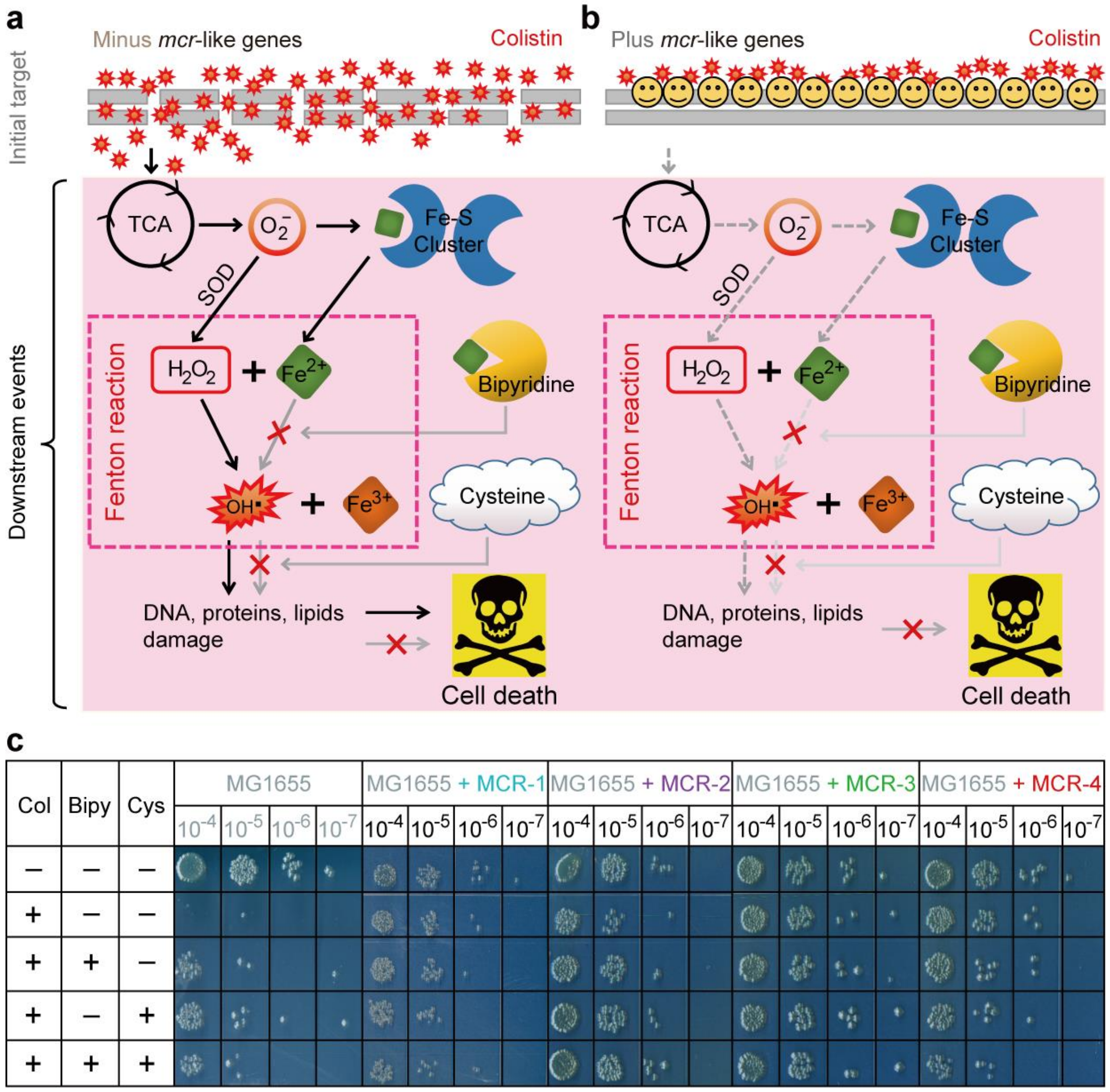

Supplementary Figure 12 A working model for MCR-mediated impairment of the hydroxyl radical death pathway in *E. coli*

a. Cartoon illustration for membrane damage triggered by the cationic antimicrobial polypeptide colistin and the resultant ROS production in *E. coli*
b. Expression of MCR-1/2/3/4 prevents the penetration of colistin into bacterial membrane and then interferes the induction of ROS in *E. coli*
c. Chemical rescue assays suggest that Fenton reaction participates into colistin-induced hydroxyl radical killing pathway in *E. coli*

The LPS-lipid A moiety refers to an initial target of colistin challenge. Bipyridine is a well-known ferric chelator, and L-cysteine is the ROS scavenger.
The model (in panels **a** & **b**) is generated with the software of Adobe Illustrator combined with PowerPoint, which is mainly adapted from Xu *et al.* [3] and Wei *et al*. [8] with permissions.

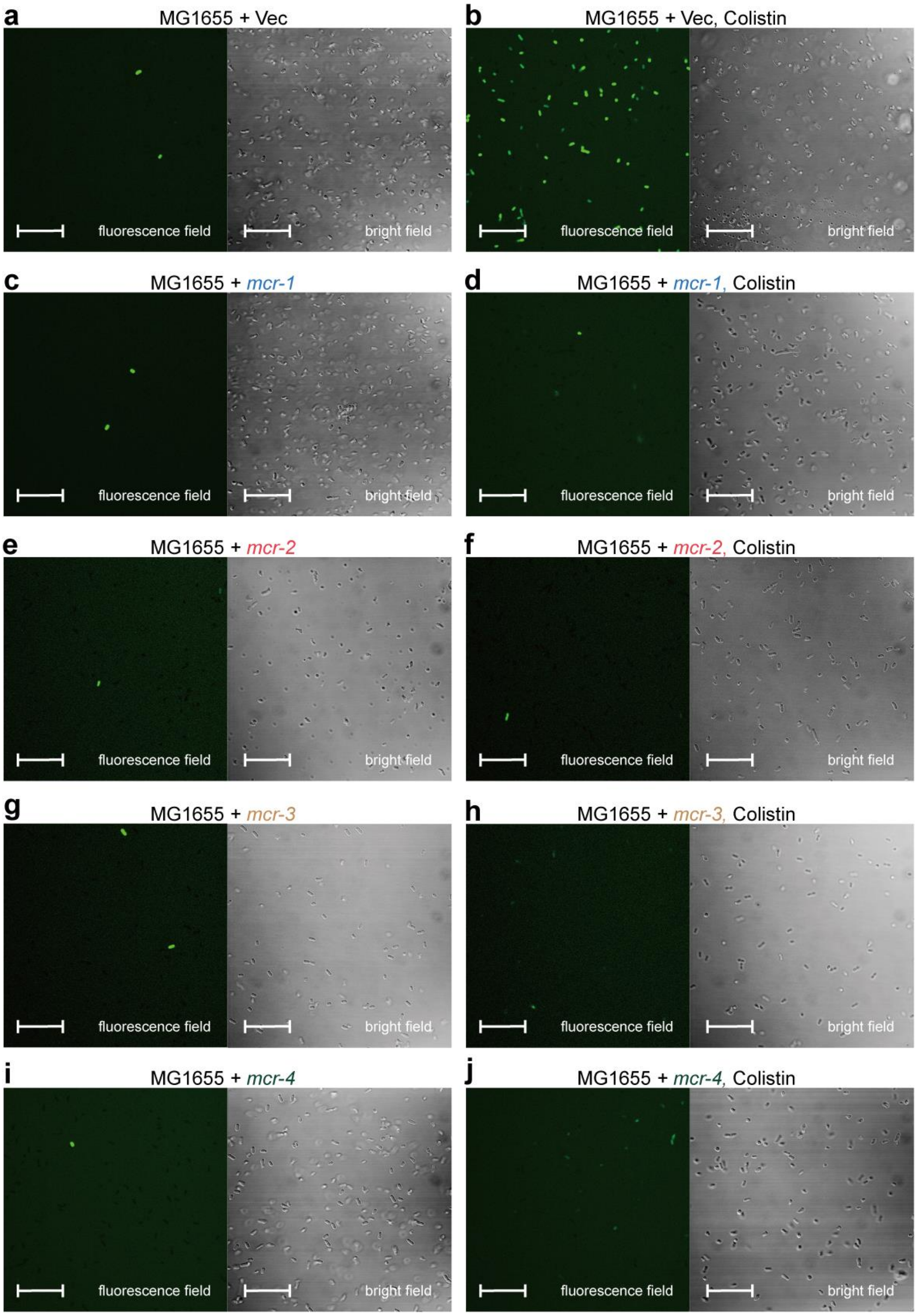

Supplementary Figure 13 ROS production is induced by colistin, but can be interfered by the expression of diversified *mcr*-like determinants

a & b Exposure of *E. coli* to colistin promotes the *in vivo* production of

hydrogen peroxide
c & d The presence of plasmid-borne MCR-1 impairs colistin-triggered accumulation of hydrogen peroxide in *E. coli*
e & f Colistin-activated production of hydrogen peroxide in *E. coli* is attenuated significantly by functional expression of MCR-2
g & h Induction of ROS in the colistin-stressed *E. coli* is decreased significantly by the presence of MCR-3
i & j Induction of ROS in the colistin-stressed *E. coli* is decreased significantly by the presence of MCR-4, which can exert an inhibitory effect on ROS production similar to MCR-1/2/3.

DCFH2-DA, the oxidant-sensitive dye, was utilized to capture the intra-cellular ROS level, because that it can be oxidized by hydrogen peroxide to give DCF, a fluorescent product.
A Zeiss LSM 510 Meta confocal laser scanning microscope (100x oil immersion objective) was applied to quantify the intensity of resultant fluorescence. Of note, hydrogen peroxides induced fluorescence is observed in green. The scale of bar is 18 μm.

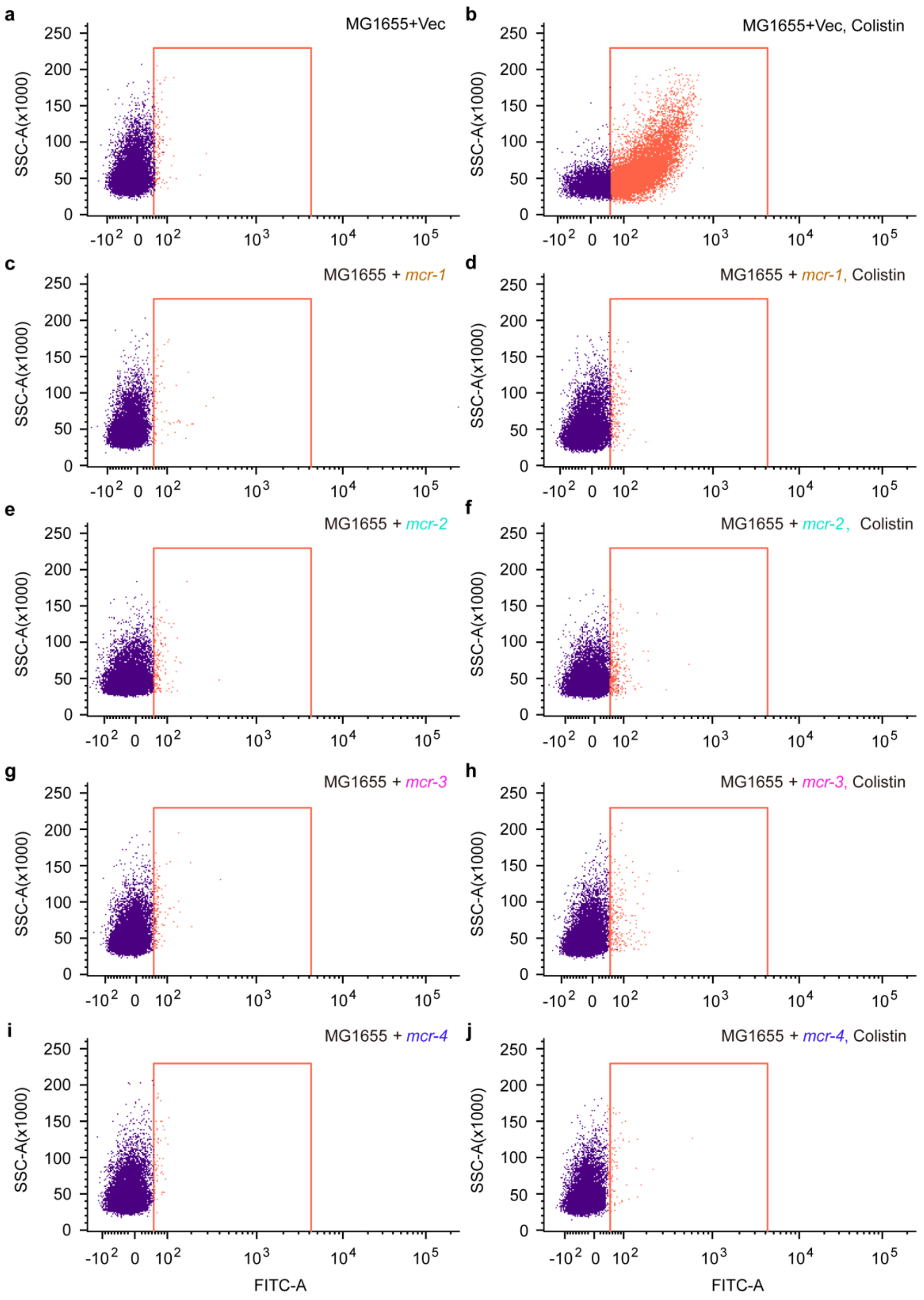

Supplementary Figure 14 Flow cytometry-aided analyses of roles of *mcr-1/2/3/4* in hydroxyl radical formation in *E. coli* stressed with colistin

a & b In the MG1655 strain with the empty vector alone, the addition of colistin accelerates the accumulation of hydroxyl radicals *in vivo*

c & d The addition of colistin cannot stimulate the *in vivo* production of ROS in *E*. *coli* carrying *mcr-1*

e & f No significant increment in bacterial ROS in the *mcr-2*-harboring MG1655 strain in the presence of colistin

g & h Functional impairment of the de novo ROS formation by the presence of MCR-4

I & j Expression of *mcr-4* prevents the penetration of colistin into MG1655 strain, thereby quenches the de novo formation of hydroxyl radicals

Designation: Vec, pBAD24.8xHis

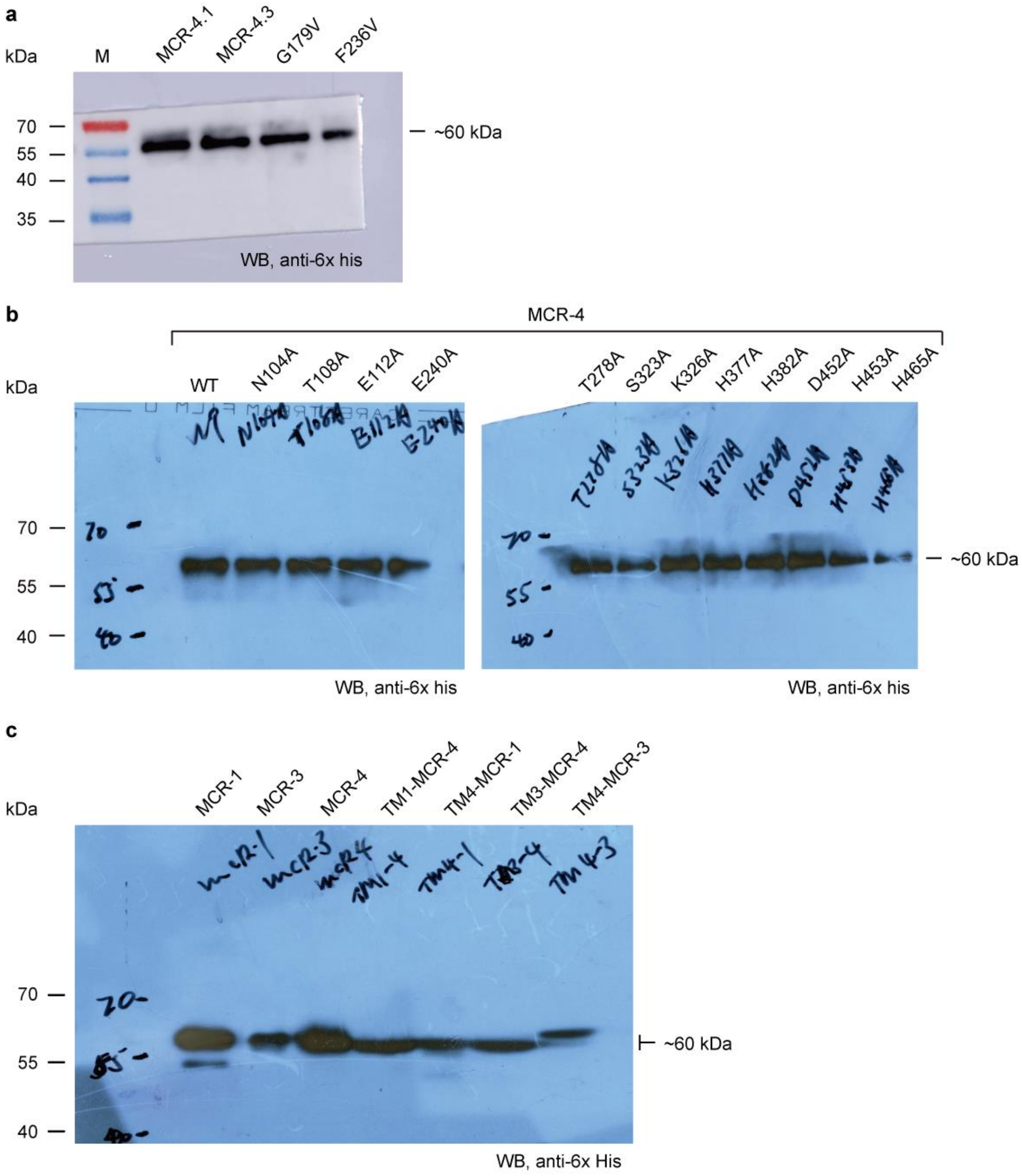

Supplementary Figure 15 Original blots used in WB analyses
a. Original blot of WB analysis in Fig. 2c
b. Original blot of WB analysis in Fig. 4e
c. Original blot of WB analysis in Fig. 5b

WB denotes western blot.

Supplementary Table 1 Strains and plasmids used in this study

Strain or plasmids	Relevant characteristics	Origins
**Strains**		
DH5α	A cloning host of *E. coli*	Lab stock
MG1655	A wild-type strain of *E. coli*	Lab stock
FYJ795	MG1655 carrying pBAD24::*mcr-1*	Lab stock
FYJ796	MG1655 carrying pBAD24	Lab stock
FYJ1126	MG1655 carrying pBAD24::*mcr-3*	Lab stock
FYJ1413	MG1655 carrying pBAD24::*mcr-4*	This work
FYJ1414	MG1655 carrying pBAD24::*mcr-4* (N104A)	This work
FYJ1415	MG1655 carrying pBAD24::*mcr-4* (T108A)	This work
FYJ1416	MG1655 carrying pBAD24::*mcr-4* (E112A)	This work
FYJ1417	MG1655 carrying pBAD24::*mcr-4* (E240A)	This work
FYJ1418	MG1655 carrying pBAD24::*mcr-4* (T278A)	This work
FYJ1419	MG1655 carrying pBAD24::*mcr-4* (S323A)	This work
FYJ1420	MG1655 carrying pBAD24::*mcr-4* (K326A)	This work
FYJ1421	MG1655 carrying pBAD24::*mcr-4* (H377A)	This work
FYJ1422	MG1655 carrying pBAD24::*mcr-4* (H382A)	This work
FYJ1423	MG1655 carrying pBAD24::*mcr-4* (D452A)	This work
FYJ1424	MG1655 carrying pBAD24::*mcr-4* (H453A)	This work
FYJ1425	MG1655 carrying pBAD24::*mcr-4* (H465A)	This work
FYJ1426	MG1655 carrying pBAD24::tm(*mcr-1*)-*mcr-4*	This work
FYJ1427	MG1655 carrying pBAD24::tm(*mcr-4*)-*mcr-1*	This work
FYJ1428	MG1655 carrying pBAD24::tm(*mcr-3*)-*mcr-4*	This work
FYJ1429	MG1655 carrying pBAD24::tm(*mcr-4*)-*mcr-3*	This work
FYJ1430	*E. coli* (Transetta DE3) carrying pBAD24.8xHis::*mcr-4*	This work
FYJ1431	*E. coli* (Transetta DE3) carrying pBAD24.8xHis::*mcr-4* (N104A)	This work
FYJ1432	*E. coli* (Transetta DE3) carrying pBAD24.8xHis::*mcr-4* (T108A)	This work
FYJ1433	*E. coli* (Transetta DE3) carrying pBAD24.8xHis::*mcr-4* (E112A)	This work
FYJ1434	*E. coli* (Transetta DE3) carrying pBAD24.8xHis::*mcr-4* (E240A)	This work
FYJ1435	*E. coli* (Transetta DE3) carrying pBAD24.8xHis::*mcr-4* (T278A)	This work
FYJ1436	*E. coli* (Transetta DE3) carrying pBAD24.8xHis::*mcr-4* (S323A)	This work
FYJ1437	*E. coli* (Transetta DE3) carrying pBAD24.8xHis::*mcr-4* (K326A)	This work
FYJ1438	*E. coli* (Transetta DE3) carrying pBAD24.8xHis::*mcr-4* (H377A)	This work

FYJ1439	*E. coli* (Transetta DE3) carrying pBAD24.8xHis*::mcr-4* (H382A)	This work
FYJ1440	*E. coli* (Transetta DE3) carrying pBAD24.8xHis*::mcr-4* (D452A)	This work
FYJ1441	*E. coli* (Transetta DE3) carrying pBAD24.8xHis*::mcr-4* (H453A)	This work
FYJ1442	*E. coli* (Transetta DE3) carrying pBAD24.8xHis*::mcr-4* (H465A)	This work
FYJ1443	*E. coli* (Transetta DE3) carrying pBAD24.8xHis*::*tm(*mcr-1*)-*mcr-4*	This work
FYJ1444	*E. coli* (Transetta DE3) carrying pBAD24.8xHis*::*tm(*mcr-4*)-*mcr-1*	This work
FYJ1445	*E. coli* (Transetta DE3) carrying pBAD24.8xHis*::*tm(*mcr-3*)-*mcr-4*	This work
FYJ1446	*E. coli* (Transetta DE3) carrying pBAD24.8xHis*::*tm(*mcr-4*)-*mcr-3*	This work
FYJ1447	DH5α carrying pBAD24.8xHis	Lab stock
FYJ1448	MG1655 carrying pBAD24*::mcr-4.3*	This work
FYJ1449	MG1655 carrying pBAD24*::mcr-4*.3 (G179V)	This work
FYJ1450	MG1655 carrying pBAD24*::mcr-4.3* (F236V)	This work
FYJ1451	*E. coli* (Transetta DE3) carrying pBAD24.8xHis*::mcr-4.3*	This work
FYJ1452	*E. coli* (Transetta DE3) carrying pBAD24.8xHis*::mcr-4.3* (G179V)	This work
FYJ1453	*E. coli* (Transetta DE3) carrying pBAD24.8xHis*::mcr-4.3* (F236V)	This work
**Plasmids**		
pBAD24	Arabinose inducible promoter-driven expression vector; Amp^R	Lab stock
pBAD24*::mcr-4*	A pBAD24 carrying the wild-type version of *mcr-4* at the two cuts of EcoRI and SalI; Amp^R	This work
pBAD24*::mcr-4*(N104A)	A pBAD24 encoding the mutant version of *mcr-4* (N104A); Amp^R	This work
pBAD24*::mcr-4*(T108A)	A pBAD24 encoding the mutant version of *mcr-4* (T108A); Amp^R	This work
pBAD24*::mcr-4*(E112A)	A pBAD24 encoding the mutant version of *mcr-4* (E112A); Amp^R	This work
pBAD24*::mcr-4*(E240A)	A pBAD24 encoding the mutant version of *mcr-4* (E240A); Amp^R	This work
pBAD24*::mcr-4*(T278A)	A pBAD24 encoding the mutant version of *mcr-4* (T278A); Amp^R	This work
pBAD24*::mcr-4*(S323A)	A pBAD24 encoding the mutant version of *mcr-4* (S323A); Amp^R	This work
pBAD24*::mcr-4*(K326A)	A pBAD24 encoding the mutant version of	This work

	mcr-4 (K326A); Amp^R	
pBAD24::*mcr-4*(H377A)	A pBAD24 encoding the mutant version of *mcr-4* (H377A); Amp^R	This work
pBAD24::*mcr-4*(H382A)	A pBAD24 encoding the mutant version of *mcr-4* (H382A); Amp^R	This work
pBAD24::*mcr-4*(D452A)	A pBAD24 encoding the mutant version of *mcr-4* (D452A); Amp^R	This work
pBAD24::*mcr-4*(H453A)	A pBAD24 encoding the mutant version of *mcr-4* (H453A); Amp^R	This work
pBAD24::*mcr-4*(H465A)	A pBAD24 encoding the mutant version of *mcr-4* (H465A); Amp^R	This work
pBAD24::tm(*mcr-1*)-*mcr-4*	A pBAD24 carrying a hybrid gene (i.e., the transmembrane region of *mcr-1* fused with the extracellular domain of *mcr-4*); Amp^R	This work
pBAD24::tm(*mcr-4*)-*mcr-1*	A pBAD24 carrying a hybrid gene (i.e., the transmembrane region of *mcr-4* fused with the extracellular domain of *mcr-1*); Amp^R	This work
pBAD24::tm(*mcr-3*)-*mcr-4*	A pBAD24 carrying a hybrid gene (i.e., the transmembrane region of *mcr-3* fused with the extracellular domain of *mcr-4*); Amp^R	This work
pBAD24::tm(*mcr-4*)-*mcr-3*	A pBAD24 carrying a hybrid gene (i.e., the transmembrane region of *mcr-4* fused with the extracellular domain of *mcr-3*); Amp^R	This work
pBAD24.8xHis	An arabinose inducible promoter-driven expression vector with C-terminal 8xHis tag, Amp^R	Lab stock
pBAD24.8xHis::*mcr-4*	A pBAD24.8xHis carrying the wild-type version of *mcr-4* at the two cuts of EcoRI and SalI; Amp^R	This work
pBAD24.8xHis::*mcr-4.3*	A pBAD24.8xHis carrying *mcr-4.3* at the two cuts of EcoRI and SalI; Amp^R	This work
pBAD24.8xHis::*mcr-4.3* (G179V)	A pBAD24.8xHis carrying the revertant of *mcr-4.3* (i.e., G179V) at the two cuts of EcoRI and SalI; Amp^R	This work
pBAD24.8xHis::*mcr-4* (F236V)	A pBAD24.8xHis carrying the revertant of *mcr-4.3* (i.e., F236V) at the two cuts of EcoRI and SalI; Amp^R	This work
pBAD24.8xHis::*mcr-4* (N104A)	A pBAD24.8xHis carrying the wild-type version of *mcr-4* (N104A) at the two cuts of EcoRI and SalI; Amp^R	This work
pBAD24.8xHis::*mcr-4* (T108A)	A pBAD24.8xHis carrying the wild-type version of *mcr-4* (T108A) at the two cuts of EcoRI and SalI; Amp^R	This work
pBAD24.8xHis::*mcr-4*	A pBAD24.8xHis carrying the wild-type	This work

(E112A)	version of *mcr-4* (E112A) at the two cuts of EcoRI and SalI; Amp^R	
pBAD24.8xHis::*mcr-4* (E240A)	A pET21a carrying the wild-type version of *mcr-4* (E240A) at the two cuts of EcoRI and SalI; Amp^R	This work
pBAD24.8xHis::*mcr-4* (T278A)	A pBAD24.8xHis carrying the wild-type version of *mcr-4* (T278A) at the two cuts of EcoRI and SalI; Amp^R	This work
pBAD24.8xHis::*mcr-4* (S323A)	A pBAD24.8xHis carrying the wild-type version of *mcr-4* (S323A) at the two cuts of EcoRI and SalI; Amp^R	This work
pBAD24.8xHis::*mcr-4* (H377A)	A pBAD24.8xHis carrying the wild-type version of *mcr-4* (H377A) at the two cuts of EcoRI and SalI; Amp^R	This work
pBAD24.8xHis::*mcr-4* (H382A)	A pBAD24.8xHis carrying the wild-type version of *mcr-4* (H382A) at the two cuts of EcoRI and SalI; Amp^R	This work
pBAD24.8xHis::*mcr-4* (D452A)	A pBAD24.8xHis carrying the wild-type version of *mcr-4* (D452A) at the two cuts of EcoRI and SalI; Amp^R	This work
pBAD24.8xHis::*mcr-4* (H453A)	A pBAD24.8xHis carrying the wild-type version of *mcr-4* (H453A) at the two cuts of EcoRI and SalI; Amp^R	This work
pBAD24.8xHis::*mcr-4* (H465A)	A pBAD24.8xHis carrying the wild-type version of *mcr-4* (H465A) at the two cuts of EcoRI and SalI; Amp^R	This work
pBAD24.8xHis::tm(*mcr-1*)-*mcr-4*	A pBAD24.8xHis carrying the transmembrane region of *mcr-1* that is fused with the extracellular domain of *mcr-4* at the two cuts of EcoRI and SalI; Amp^R	This work
pBAD24.8xHis::tm(*mcr-4*)-*mcr-1*	A pBAD24.8xHis carrying the transmembrane region of *mcr-4* that is fused with the extracellular domain of *mcr-1* at the two cuts of EcoRI and SalI; Amp^R	This work
pBAD24.x8His::tm(*mcr-3*)-*mcr-4*	A pBAD24.8xHis carrying the transmembrane region of *mcr-3* that is fused with the extracellular domain of *mcr-4* at the two cuts of EcoRI and SalI; Amp^R	This work
pBAD24.8xHis::tm(*mcr-4*)-*mcr-3*	A pBAD24.8xHis carrying the transmembrane region of *mcr-4* that is fused with the extracellular domain of *mcr-3* at the two cuts of EcoRI and SalI; Amp^R	This work

Supplementary Table 2 Oligonucleotides used in this study

Primers	Primer sequences (5’--3’)
pBAD24-*mcr-4*-F(EcoRI)	5’-CG GAATTC GTG ATT TCT AGA TTT AAG ACG T-3’
pBAD24-*mcr-4*-R(SalI)	5’-GC GTCGAC CTA ATA CCT GCA AGG TGC AA-3’
pBAD24.8xHis-*mcr-4*-F(EcoRI)	5’-CG GAATTC GTG ATT TCT AGA TTT AAG ACG T-3’
pBAD24.8xHis-*mcr-4*-R(SalI)	5’-GC GTCGAC ATA CCT GCA AGG TGC AAA AAT-3’
pBAD24-*mcr-1*-F(EcoRI)	5’-CCG GAATTC ATG ATG CAG CAT ACT TCT GTG T-3’
pBAD24-*mcr-1*-R(SalI)	5’-ACGC GTCGAC TCA GCG GAT GAA TGC GGT GC-3’
pBAD24-*mcr-3*-F(EcoRI)	5’-CCG GAATTC ATG CCT TCC CTT ATA AAA ATA AAA A-3’
pBAD24-*mcr-3*-R(SalI)	5’-ACGC GTCGAC TTA TTG AAC ATT ACG ACA TTG ACT-3’
pBAD24.8xHis-*mcr-1*-F (EcoRI)	5’-CCG GAATTC ATG ATG CAG CAT ACT TCT GTG T-3’
pBAD24.8xHis-*mcr-1*-R(SalI)	5’-ACGC GTCGAC GCG GAT GAA TGC GGT GCG GT-3’
pBAD24.8xHis-*mcr-3*-F (EcoRI)	5’-CCG GAATTC ATG CCT TCC CTT ATA AAA ATA AAA A-3’
pBAD24.8xHis-*mcr-3*-R(SalI)	5’-ACGC GTCGAC TTG AAC ATT ACG ACA TTG ACT GAA-3’
pBAD24.8His-*mcr-4.3*-F(EcoRI)	5’-CG GAATTC GTG ATT TCT AGA TTT AAG ACG T-3’
pBAD24.8His-*mcr-4.3*-R(SalI)	5’-GC GTCGAC ATA CCT GCA AGG TGC AAA AAT-3’
mcr-4.3 (G179V)-F	5’-CT GCA TTT **GTT** CGA AAC AAC AGT GAG TTA AGG CG-3’
mcr-4.3 (G179V)-R	5’-GTT TCG **AAC** AAA TGC AGC ATA ATC TTG ATA GTA AAA A-3’
mcr-4.3 (F236V)-F	5’-TTA GTG **GTT** GTT GTG GGT GAA ACT GCG CGC TC-3’
mcr-4.3 (F236V)-R	5’-CC CAC AAC **AAC** CAC TAA TAA GTT AGG TTT AGT GTT CGG G-3’
mcr-1-TM-R	5’-GAA CAA ATG CAG CAT AAT CTT GGG CAT AAT GAC TGC TGA ACG-3’
mcr-4-OS-F	5’-GGA TAG TCG CCT TTT TTT ACT ATA GTT TCT TTC GCG TGC ATA AG-3’
mcr-3-TM-R	5’-GAA CAA ATG CAG CAT AAT CTT GAT AGT ATA GTG CTG CAA TAA CC-3’
mcr-4-OS-F	5’-GGA TAG TCG CCT TTT TTT ACT ATC AAG ATT ATG TGT CAG TGG GG-3’
mcr-4-TM-R	5’-CTT ATG CAC GCG AAA GAA ACT ATA GTA AAA AAA GGC GAC TAT CC-3’
mcr-1-OS-F	5’-CGT TCA GCA GTC ATT ATG CCC AAG ATT ATG CTG CAT TTG TTC-3’
mcr-4-TM-R	5’-CCC CAC TGA CAC ATA ATC TTG ATA GTA AAA AAA GGC GAC TAT CC-3’
mcr-3-OS-F	5’-GGT TAT TGC AGC ACT ATA CTA TCA AGA TTA TGC

	TGC ATT TGT TC-3’
mcr-4 (N104A)-F	5’-GAT AGA A**GC A**AC GTT TCA AAC ACA TCC TGC TG-3’
mcr-4 (N104A)-R	5’-GAA ACG T**TG C**TT CTA TCA TGC CGT AGT CAA ACA CG-3’
mcr-4 (T108A)-F	5’-GTT TCA A**GC A**CA TCC TGC TGA AGC ATT GAT GT-3’
mcr-4 (T108A)-R	5’-CAG GAT G**TG C**TT GAA ACG TGT TTT CTA TCA TGC C-3’
mcr-4 (E112A)-F	5’-TCC TGCT**GC A**GC ATT GAT GTA TGT AAA TCT TGC ATC-3’
mcr-4 (E112A)-R	5’-TCA ATG C**TG C**AG CAG GAT GTG TTT GAA ACG TG-3’
mcr-4 (E240A)-F	5’-TGT TGT GGG T**GC A**AC TGC GCG CTC AAT GAG CT-3’
mcr-4 (E240A)-R	5’-CAG T**TG C**AC CCA CAA CAA CCA CTA ATA AGT TAG G-3’
mcr-4 (T278A)-F	5’-CC**G CA**G CGG TGT CTC TAC CCT GTA TGT TTT CA -3’
mcr-4 (T278A-R	5’-TAG AGA CAC CGC **TGC** GGC CGT GCC GCA TGA GCT-3’
mcr-4 (S323A)-F	5’-GAT AAT GAT **GCA** GGC TGT AAA GGT GTG TGT GAT CA-3’
mcr-4 (S323A)-R	5’-CAG CC**T GC**A TCA TTA TCA AAC CAC TGT ACT TTT ATA CC-3’
mcr-4 (K326A)-F	5’-ATT CTG GCT GT**G CA**G GTG TGT GTG ATC AGG TTG AAA AT-3’
mcr-4 (K326A)-R	5’-ACC **TGC** ACA GCC AGA ATC ATT ATC AAA CCA CT-3’
mcr-4 (H377A)-F	5’-G**GC A**AT CAT TGG TAG TCA TGG ACC AAC TTA TT-3’
mcr-4 (H377A)-R	5’-GAC TAC CAA TGA T**TG C**CA AAA AAA TTA CTG TAT CTT GAC TTG GTG-3’
mcr-4 (H382A)-F	5’-TGG TAG T**GC A**GG ACC AAC TTA TTA TCT TAG ATA CCC G-3’
mcr-4 (H382A)-R	5’-TTG GTC C**TG C**AC TAC CAA TGA TAT GCA AAA AAA TTA CTG-3’
mcr-4 (D452A)-F	5’-ATC TCT CT**G CA**C ATG GTG AGT CTT TGG GTG AAA A-3’
mcr-4 (D452A)-R	5’-ACC ATG **TGC** AGA GAG ATA CAG CAT TGC AGT ATC G-3’
mcr-4 (H453A)-F	5’-TGA C**GC A**GG TGA GTC TTT GGG TGA AAA GGG CA-3’
mcr-4 (H453A)-R	5’-AAG ACT CAC C**TG C**GT CAG AGA GAT ACA GCA TTG CAG TAT C-3’
mcr-4 (H465A)-F	5’-GTA TTT A**GC A**GG TGC GCC CTA TAG TAT TGC AC-3’
mcr-4 (H465A)-R	5’-GCG CAC C**TG C**TA AAT ACA TGC CCT TTT CAC CCA-3’

*The underlined letters in italics denote restrictions sites, and the letters in bold refer to the point mutations.

Supplementary References

1. Chavda, B. et al. Coidentification of *mcr-4.3* and *bla*$_{NDM-1}$ in a clinical *Enterobacter cloacae* isolate from China. *Antimicrob Agents Chemother* **62**, e00649-00618 (2018).
2. Ding, S. et al. Discovery of multi-drug resistant, MCR-1 and ESBL-coproducing ST117 *Escherichia coli* from diseased chickens in Northeast China. *Sci Bull (Beijing)* **63**, 1059-1066 (2018).
3. Xu, Y. et al. Spread of MCR-3 colistin resistance in China: An epidemiological, genomic and mechanistic study. *EBioMedicine*, pii: S2352-3964(2318)30270-30276 (2018).
4. Xu, Y. et al. An evolutionarily conserved mechanism for intrinsic and transferable polymyxin resistance. *mBio* **9**, e02317-02317 (2018).
5. Xu, Y., Lin, J., Cui, T., Srinivas, S. & Feng, Y. Mechanistic insights into transferable polymyxin resistance among gut bacteria. *J Biol Chem* **293**, 4350-4365 (2018).
6. Anandan, A. et al. Structure of a lipid A phosphoethanolamine transferase suggests how conformational changes govern substrate binding. *Proc Natl Acad Sci U S A* **114**, 2218-2223 (2017).
7. Sun, J., Zhang, H., Liu, Y.H. & Feng, Y. Towards understanding MCR-like colistin resistance. *Trends Microbiol* **26**, 794-808 (2018).
8. Wei, W. et al. Defining ICR-Mo, an intrinsic colistin resistance determinant from *Moraxella osloensis*. *PLoS Genet* **14**, e1007389 (2018).
